# Historical Differentiation and Recent Hybridization in Natural Populations of the Nematode-Trapping Fungus *Arthrobotrys oligospora* in China

**DOI:** 10.3390/microorganisms9091919

**Published:** 2021-09-09

**Authors:** Duanyong Zhou, Jianping Xu, Jianyong Dong, Haixia Li, Da Wang, Juan Gu, Ke-Qin Zhang, Ying Zhang

**Affiliations:** 1 State Key Laboratory for Conservation and Utilization of Bio-Resources in Yunnan, Key Laboratory for Southwest Microbial Diversity of the Ministry of Education, Yunnan University, Kunming 650032, China; zhouduanyong1112@163.com (D.Z.); djy920506@163.com (J.D.); 17806253435@163.com (H.L.); wd17805050566@163.com (D.W.); 2 School of Life Science, Yunnan University, Kunming 650032, China; gj15094221836@163.com; 3 School of Biology and Chemistry, Xingyi Normal University for Nationalities, Xingyi 562400, China; 4 Department of Biology, McMaster University, Hamilton, ON L8S 4K1, Canada

**Keywords:** short tandem repeat, genetic differentiation, unique alleles, non-random recombination, phenotypic variation, nematicidal activity

## Abstract

Maintaining the effects of nematode-trapping fungi (NTF) agents in order to control plant-parasitic nematodes (PPNs) in different ecological environments has been a major challenge in biological control applications. To achieve such an objective, it is important to understand how populations of the biocontrol agent NTF are geographically and ecologically structured. A previous study reported evidence for ecological adaptation in the model NTF species *Arthrobotrys oligospora*. However, their large-scale geographic structure, patterns of gene flow, their potential phenotypic diversification, and host specialization remain largely unknown. In this study, we developed a new panel of 20 polymorphic short tandem repeat (STR) markers and analyzed 239 isolates of *A. oligospora* from 19 geographic populations in China. In addition, DNA sequences at six nuclear gene loci and strain mating types (MAT) were obtained for these strains. Our analyses suggest historical divergence within the *A. oligospora* population in China. The genetically differentiated populations also showed phenotypic differences that may be related to their ecological adaptations. Interestingly, our analyses identified evidence for recent dispersion and hybridization among the historically subdivided geographic populations in nature. Together, our results indicate a changing population structure of *A. oligospora* in China and that care must be taken in selecting the appropriate strains as biocontrol agents that can effectively reproduce in agriculture soil while maintaining their nematode-trapping ability.

## 1. Introduction

Plant-parasitic nematodes (PPNs), especially the root-knot nematodes in the genus *Meloidogyne*, are widespread pests that cause crop yield losses worth more than US $157 billion worldwide each year [1]. For decades, the control of *Meloidogyne* spp. has heavily relied on chemical nematicides. However, resistance to chemical nematicides has emerged, and the environmental and human health impacts of nematicide residues are becoming increasingly recognized [2]. Therefore, currently available chemical nematicides are being phased out, and an increasing number of biocontrol agents are being introduced to help control PPNs [3].

At present, about 700 fungal species are known to be capable of attacking living nematodes (juveniles, adults, and eggs). These fungi are taxonomically diverse but traditionally divided into five groups based on the mechanisms by which they attack nematodes: (i) nematode-trapping fungi (NTF), (ii) endoparasitic fungi, (iii) egg- and cyst-parasitic fungi, (iv) toxin-producing fungi, and (v) fungi with special nematode-attacking devices [4]. Among these groups, the broad adaptability and flexible lifestyles of NTFs make them ideal agents for controlling PPNs [5,6,7]. NTFs are abundantly distributed in a broad range of habitats, especially in temperate agricultural pastures, coniferous leaf litters, and coastal vegetations [8]. Among the NTFs, *A. oligospora* has generally been considered to be the most common nematode predator in temperate soils [9]. In soil environments, *A. oligospora* naturally encounters a broad range of nematodes and behaves as a generalist predator with the characteristic ability of forming adhesive trapping nets when its mycelia are in contact with nematodes. Unlike endoparasitic fungi, NTFs are able to grow saprophytically. However, how they maintain and balance their saprophytic and predatory lifestyles are not known. In addition, how geographic populations interact with each other and how ecological factors impact population dynamics remain poorly understood. Such knowledge is important not only for understanding the diversity and evolution of these fungi but also for identifying the most appropriate isolate(s) for commercial biological-control applications [3,6].

Most ecological studies of nematophagous fungi have been restricted to surveys on their geographical and seasonal distributions, including examining the effects of abiotic (e.g., soil conditions) and biotic (mainly nematode density) factors on their distributions [8,10,11,12,13,14,15]. Though molecular techniques have increasingly been used to examine phylogenetic relationships among nematode-trapping Orbiliales [16,17,18,19,20] and to investigate the molecular genetics of fungus–nematode interactions [21,22,23,24,25,26,27,28,29], they have scarcely been used to study the patterns of genetic variation present in NTF populations, including the spatial and temporal distributions of genotypes in specific species. The authors of an earlier study [30] analyzed 97 *A. oligospora* strains from several geographic locations and ecological niches in China using DNA sequences at three gene fragments: *its* (internal transcribed spacer region of the ribosomal RNA), *tub* (β-tubulin), and *rpb2* (RNA polymerase II subunit). That study identified a large number of unique alleles and genotypes, as well as their limited geographic distributions, consistent with a large effective population size of *A. oligospora* and its potential for genetic differentiation among geographic populations, likely driven by ecological adaptation [30]. Further analyses of more samples using restriction fragment length polymorphism (RFLP) genotyping revealed a similar pattern [31]. Recently, the heritability of *A. oligospora* phenotypic variation in the response to nematodes was assessed by analyzing the genetic variation of genomic SNPs using 18 wild isolates [21]. However, due to limited sampling at most geographic locations and/or the small number of molecular loci used, *A. oligospora’s* overall patterns of genetic variations, the relationships between genetic variations and phenotypic variations, and its mode of reproduction remain to be fully described.

For this study, we conducted a broad range of sampling on the model NTF species *A. oligospora* in China. These samples were analyzed with a panel of 20 short tandem repeat (STR or microsatellite) markers newly developed for this study. Combined with the DNA sequences of six housekeeping genes (*its*, *tub*, *tef-1*, *rpb2*, mitogen-activated protein kinase (*mapk*), and subtilisin-like serine protease (*sp*)) and mating type (MAT) genes (*mat 1-1* and *mat 1-2*), the population structure, phylogenetic relationships, and mode of reproduction of the nematode-trapping hyphomycete *A. oligospora* in nature were investigated. In addition, we analyzed the patterns of dispersal and potential ecological niche adaptations of strains in China. Furthermore, representative strains from each of the robust phylogenetic lineages [30] were used to explore the intraspecific differentiation by studying the size and shape of the conidia, the growth rate of the colony under the experimental conditions, and their nematicidal activity. Our analyses revealed evidence for historical differentiation, as well as recent dispersion and hybridization. The implications of our results regarding the application of *A. oligospora* as a biocontrol agent against PPNs in agricultural fields are discussed.

## 2. Materials and Methods

### 2.1. Strains

**Figure 1:**
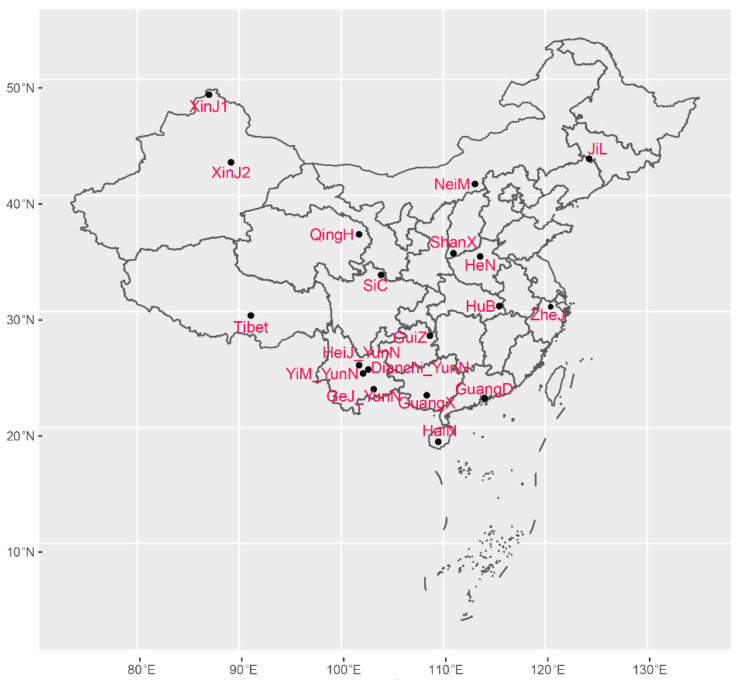
Geographic distribution of all the *A. oligospora* samples collected in China. Note: the full names of the sampling sites are indicated in Table 1.

This study analyzed a total of 239 strains of *A. oligospora*, including 104 previously analyzed strains [30,31] and 135 strains newly isolated from seven provinces. These isolates were obtained from agricultural and forest soils from 19 geographic populations in 14 provinces, covering seven large regions in China; the detailed geographic information of sampling sites and population size are given in Figure 1 and Table 1. The isolation and morphological identification of *A. oligospora* were based on the standard method described by Zhang et al. [31]. Briefly, for each sample, 1–2 g of soil/water were sprinkled onto sterile corn meal agar (CMA; 20 g of cornmeal, 18 g of agar, and enough water to reach a final volume of 1000 mL) and then free-living *Panagrellus redivius* nematodes were added onto the culture plate and inoculated at room temperature. Each sample was screened three independent times, resulting in 1500 agar plates for a total of 500 soil/water samples. After one month, nematodes were examined using a dissecting microscope. Single spores were directly isolated from the mycelia that trapped nematodes and then cultivated on a CMA Petri dish at 25 °C. The taxonomic characters were examined from cultures on CMA at 7–14 days after inoculation. The initial identification of *A. oligospora* was based on species-specific microscopic and macroscopic colony morphological features. The sizes of conidia and conidiophores were observed using an Olympus BX51 microscope (Olympus Corporation, Tokyo, Japan).

### 2.2. Identification of Tandem Repeat Loci and Development of Amplification Primers

STRs (or microsatellites) have been extensively used for molecular ecology and population genetic studies due to their high-level polymorphisms and high reproducibility in eukaryotic genomes [32,33,34,35,36]. In this study, the STR markers were developed based on the genome sequence of *A. oligospora* strain ATCC24927 (GenBank accession ADOT00000000), using the MISA software (Available online: https://webblast.ipk-gatersleben.de/misa/ (accessed on 12 April 2021)). From the candidate STR markers in the genome, we selected three di-, tri-, and tetranucleotide repeats based on loci with the highest repeat numbers and an interruption (max difference: 2 SSRs) of 100 bp within the boundaries of potential PCR primer regions [37]. Primer pairs were designed from flanking sequences of each STR locus (Available online: http://pgrc.ipk-gatersleben.de/misa/primer3.html (accessed on 13 April 2021)), and primers with lengths of 20–23 bp, a T_m_ value about 60 °C, and product sizes in the 150–300 bp range were selected for further screening. For selected primer pairs, fluorescent tags were added to the forward primers (Table 2). The fluorescent PCR products were examined via capillary electrophoresis assay on an Applied Biosystems (ABI 3730xl) sequencer (Applied Biosystems, Foster City, CA, USA). The GeneMapper 4.1 software (Available online: http://www.appliedbiosys-tems.com/Genemapper (accessed on 15 June 2021)) was employed to determine the polymorphism information content (PIC) for each STR marker.

### 2.3. DNA Extraction, PCR Amplification and Sequencing

Genomic DNA was extracted from the mycelia collected from single-spore cultures growing on cellophane membrane on potato dextrose agar (PDA) according to the modified CTAB method [38]. Fragments of the following six genes were studied: *its* [39], *tub* [40], *rpb2* [30], translation elongation factor 1 alpha (*tef-1*), mitogen-activated protein kinase (*mapk*), and subtilisin-like serine protease (*sp*) [19]. Similarly, mating type genes *mat1-1* and *mat1-2* were amplified using the primer pairs MAT1F (5′CCAGAGCAAAGTTCACGG3′) and MAT1R (5′GTAGGAGGTTCTAGGGCG3′), MAT2F (5′GCTTGCCTTCTGTTTACGG3′) and MAT2R (5′GTTTATTGAGAGGTGGGTC3′). The obtained *its* sequences were compared with known sequences of *A. oligospora* using BLAST to confirm their species identification first for all candidate strains [41].

PCR reactions were performed using 1 µL of genomic DNA template in a 50 µL PCR mixture (1.5 units of Taq DNA polymerase, 10 × PCR buffer (100 mM KCl; 200 mM Tris-HCl, pH 8.8; 100 mM (NH4)_2_SO_4_; 20 mM MgSO_4_; 1% Triton X-100; 1 and mg/mL BSA), 1.5 mM MgCl_2_, 2.5 mM dNTPs, and 1.5 mM primers) under the following amplifying conditions: 1 min of initial denaturation at 94 °C, followed by 30 cycles of 1 min of denaturation at 94 °C, 1 min of primer annealing at 50 °C, 1.5 min of extension at 72 °C, and a final extension period of 10 min at 72 °C. The PCR products were visualized under UV light exposure, and the purified fragments were directly sequenced on both strands with the same primers that were used for amplification.

### 2.4. Data Analysis

The genotyping of *A. oligospora* isolates was performed with both selected STR loci and single nucleotide polymorphic (SNP) loci from multilocus sequence typing (MLST). Alleles at the each STR locus were combined with SNPs to generate the multilocus genotype for each strain. All genotypes were entered into Microsoft Excel to conduct population genetic analyses by GenAlEx version 6.1 (The Australian National University, Canberra, Australia) [42]. For the LOCPRIOR model with admixture and correlated allele frequencies, the program STRUCTURE version 2.3.3 (Stanford University, Stanford, CA, USA) [43] was used to explore the number of genetic clusters (K) occurring in the sample. A total of 10 replicates were performed of each simulation for K = 1–12, with a burnin of 10,000 and an MCMC of 100,000 iterations for the best fixed value of K. Furthermore, a minimum spanning tree was constructed with default settings based on STR genotypes (BIONUMERICS 8.0, Applied Maths, Sint-Martens-Latem, Belgium) to investigate the genetic relationship between isolates from China.

It has been reported that the corresponding teleomorph (sexual stage) of *A. oligospora* is *Orbilia auricolor* [44]. Interestingly, three other morphologically distinct anamorphs have been linked to the same teleomorphic species [45]. Theoretically, there are 15 different possible links between sexual and asexual morphs [46]. Thus far, the sexual stage has been rarely reported in laboratory cultures or nature for *A. oligospora*. Thus, its importance in nature remains largely unknown. In a previous study, a population in a stressful environment was shown to have significant evidence of recombination, likely due to sexual reproduction [31]. Here, to examine the reproductive mode in natural populations of this species, the distributions of mating type idiomorphs (*mat1-1* and *mat1-2*) were analyzed from our *A. oligospora* strains and populations, and the following two complementary tests were conducted. First, we calculated the index of association (I_A_) and rBarD. The null hypothesis for I_A_ is that there is random association (recombination, linkage equilibrium) among alleles at different loci [47], and I_A_ was standardized by the number of loci, with the rBarD algorithm adjusting for the numbers of loci. In the second test, we calculated the proportion of pairwise loci that were phylogenetically compatible (PrC). PrC is an indicator of recombination at the population level. In contrast, a lack of phylogenetic incompatibility implies asexual reproduction. Both tests were conducted using MultiLocus version 1.3 (Department of Biology, Imperial College, Ascot, UK) [47].

### 2.5. Intraspecific Phenotypic Variation

#### 2.5.1. Comparison of Mycelial Growth and Conidial Shape

In our previous study, three divergent lineages (cryptic species) were suggested for samples from China, and these were also included in this study [30]. The comparisons of our intraspecific phenotypic variation were all based on divergence of the three lineages. To compare the growth rate and conidial yield of samples from the three different lineages (A–C) under different nutritional and temperature conditions, 7 mm diameter hyphal disks punched from the edges of plate colonies were inoculated onto three agar media (PDA; CMA; and tryptone, yeast extract and glucose agar (TYGA)), with each treatment incubated at three temperatures (25, 28, and 30 °C, respectively) for 3–7 days. The mycelial growth rate and colony morphology were examined and quantified at specific time intervals [48]. The aforementioned hyphal disks were also incubated on corn meal yeast extract agar (CMY) plates for 14 days at 28 °C. The conidial yield of each strain was then measured as previously described [49]. The morphological descriptions and photographs of hyphal and conidial morphology were observed and measured using fresh specimens growing on CMA. Both the lengths and widths of each conidium were observed and measured with an Olympus BX51 microscope with differential interference contrast. Measurement data were based on 50 random conidia of each strain, and the length–width ratios were used for comparative analyses among samples.

#### 2.5.2. Trap Formation and Bioassay

The *Caenorhabditis elegans* employed in this study were cultured on nematode growth media (NGM) with *Escherichia coli* (OP50) as food source and maintained following standard procedures [50].

To investigate their nematode-trapping abilities, freshly harvested conidia (2 × 10^4^) of strains of *A. oligospora* from the three different lineages were evenly spread on water agar (WA) and incubated at 28 °C for 3–4 days. About 200 nematodes were added to each WA plate to induce trap formation. Traps and captured nematodes were examined and quantified (per plate) under a microscope at 12 h intervals according to our previous observation of trap formation: the appearance of immature traps and mature ones could be found at every 12 h after nematode induction. Nematodes crawling on the wall of the plate were excluded because they were not in contact with the fungal hyphae. The experiments were performed in triplicate.

#### 2.5.3. Nematicidal Activity of Fermentation Broth

To determine the potential nematicidal activity of fermented broth of each isolate, 1 cm diameter hyphal disks punched from the edges of plate colonies were inoculated into potato-dextrose broth medium (PDB) for 15 d at 28 °C and 180 rpm. The fermentation broth was then centrifuged at 11,000 rpm for 30 min. The supernatants were collected, and their nematicidal effects were measured in 24-well microtiter plates. In each well, 1 mL of a supernatant was mixed with 5 μL of live nematodes (at 50–100 L2 nematodes per μL). At room temperature, the number of dead nematodes and the total amount were counted at 2, 4, 8, 12, 24, 48, and 72 h, respectively. A nematode was considered dead if it was stiff or bent irregularly for a long time. A PDB medium and an M9 buffer were used as controls. The mortality of nematodes was defined as the ratio of dead nematodes over the tested nematodes, and the corrected mortality rate was calculated by the ratio of tested mortality minus those of controls over “1-mortality of controls”.

#### 2.5.4. Statistical Analysis

To compare the differences among strains and populations, we calculated the means and standard deviations for each treatment. A one-way analysis of variance followed by Tukey’s multiple comparison test (when necessary) were used to analyze data, and *p* values < 0.05 were considered statistically significant. All statistical analyses were conducted using IBM SPSS statistical software V22.0 (IBM, Armonk, NY, USA).

## 3. Results

### 3.1. Genetic Diversity of the Chinese Samples of A. oligospora Detected by STRs and MLST

Based on 200 initially designed STR primer pairs from the genome of *A. oligospora* (ATCC 24927), we selected 20 to genotype strains of *A. oligospora.* Information about these 20 STR markers in our sample is shown in Table 2. A previous study classified the polymorphism level of STR markers based on PIC values into low (PIC < 0.25), medium (0.5 > PIC > 0.25), and high (PIC > 0.5) [51]. Based on this grouping, the 20 STR markers we developed for *A. oligospora* were found to have a moderate to high level of polymorphism, with 13 having high polymorphism, six having medium polymorphism, and only one having a low PIC value (Table 2). The highest discriminatory power value for a single locus was obtained with A191, with a gene diversity value of 0.87 and 21 different alleles in our sample (Table 1 and Table 2).

**Figure 2:**
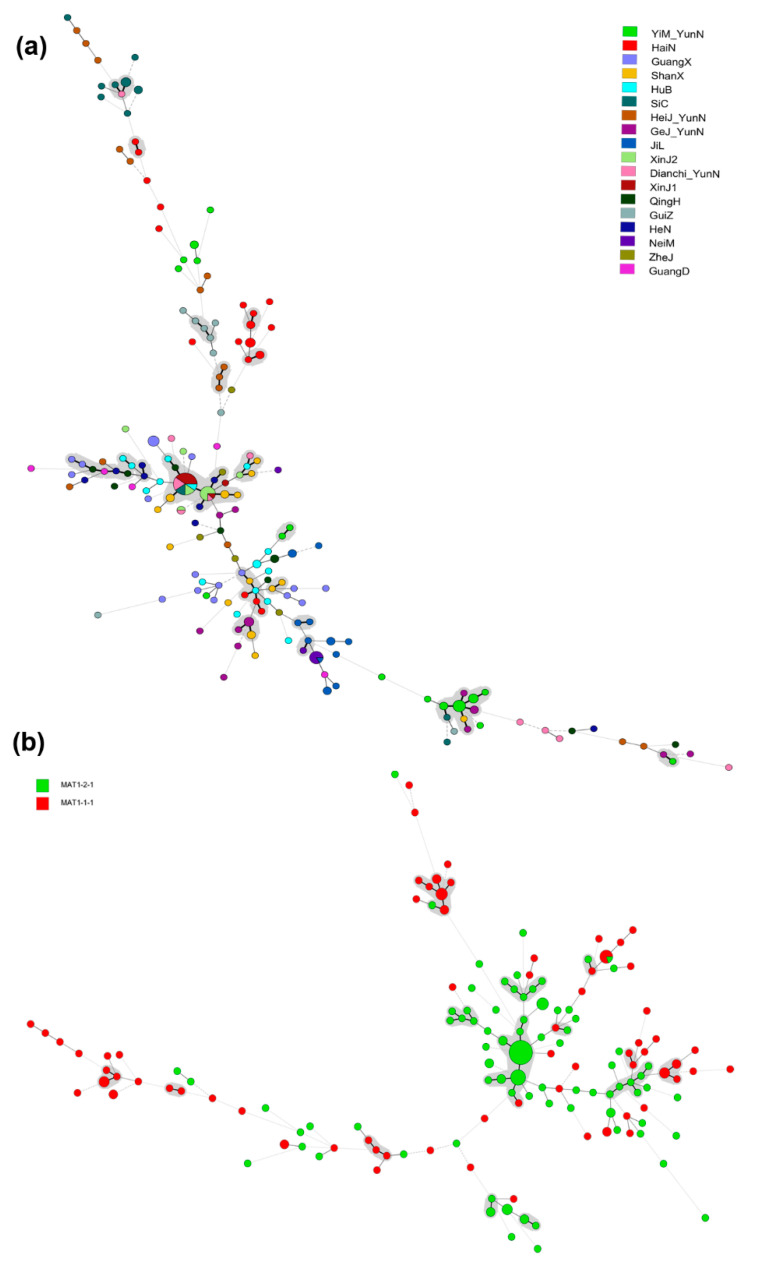
Minimum spanning tree (MST) showing the genotypic relationship (**a**) among different geographical populations and (**b**) between different mating types containing *A. oligospora* isolates. Notes: Each circle corresponds to a unique genotype, and the size of the circle proportionally represents the number of isolates with that genotype. Connecting lines correspond to the number of differences between the genotypes. Short bold line, 1 difference; black line, 2 differences; long grey line, 3 differences; dotted line, 4 or more differences.

Among the 239 *A. oligospora* isolates from 19 different geographical populations, a total of 188 alleles and 178 multilocus genotypes were found based on the 20 STR loci. The number of alleles range from 3 to 21, with an average of 9.4 alleles per locus. Of the 188 alleles, 140 were shared between at least two of the 19 geographical populations in China, the remain 48 alleles were found only in one geographical population each (Table 1). The 19 geographical populations also differed in their total numbers of alleles, which ranged from 21 (Kanas Lake, Xinjiang) to 88 (Heijing, Yunnan). Except for their absence in three geographic populations (Inner Mongolia, Qinghai, and Kanas Lake, and Xinjiang populations), private alleles were found in all other 16 populations (Table 1). Of the total 178 STR multilocus genotypes, only four were shared by two or more geographical populations, and the other 174 were only found in one geographical population each. The most shared genotype was found among two populations from Xinjiang and three populations from Dianchi, Yunnan, Sichuan, and Hubei. To better visualize the relationships among strains from the 19 geographic populations, a minimum spanning tree (MST) was constructed based on their STR genotypes. Figure 2a shows information on the geographic origins of the strains superimposed on the STR genotype relationships. Interestingly, strains from many geographic locations were often intermixed with each other (Figure S1). However, samples from Hainan and Southwestern China (Yunnan, Sichuan, and Tibet provinces) formed several tight clusters on the minimum spanning tree, consistent with both localized genetic differentiation and long-distance dispersal among geographic populations in China.

The length of the combined sequences of the six gene was 2106 bp, with individual lengths ranging from 184 to 465 bp. Among the 2106 aligned nucleotides, 125 were polymorphic among the 239 strains of *A. oligospora,* and the percentages of nucleotides being polymorphic at each locus ranged from 0.0243 (*tef-1*) to 0.0995 (*mapk*), with an average 0.0594 (125/2106) (Table S1). Here, we found no clear correlation between the length of the sequenced gene fragment and the number of polymorphic nucleotide sites (R = 0.720; *p* = 0.106) among these six sequenced fragments. Based on MLST, a total of 59 multilocus genotypes were found among the 239 isolates. Among these 59 multilocus genotypes, 47 were only found in one geographical population each, while the remaining 12 were shared among geographic populations. Of the 59 MLST genotypes, Genotype #23 was the most frequently found at 29.7% (71/239), and it was found in 13 of the 19 geographic populations. The second and third most frequent MLST genotypes were Genotype #7 (9.2%; 22/239) and Genotype #1 (7.1%; 17/239), which were found in five and seven geographic populations, respectively. Except for populations in Zhejiang, Jilin, Kanas Lake and Urumqi Botanical Garden from Xinjiang, Guangxi, Gejiu in Yunnan, and Sichuan, private genotypes were found in 12 geographic populations (Table 3). Population from Yimen in Yunnan had the most private genotypes (*n* = 10), followed by Hainan and Heijing (Yunnan) populations, both containing seven private genotypes. Among the 19 geographic populations, the diversity of multilocus genotypes ranged from 0 to 0.981. Overall, the Heijing, Yunnan population had the highest genotypic diversity (0.981), followed by Qinghai (0.956).

### 3.2. Genetic Differentiation and Population Structure

To assess the relationships among the 19 *A. oligospora* geographic populations from China, we analyzed the genetic differentiation of these isolates by using the STR and MLST types of molecular markers (SNPs at all loci). After clonal correction, the sample sizes in the STR and MLST datasets were 186 and 100, respectively. The populations of Jilin and Xinjiang had only one genotype in the MLST dataset and thus could not be used for further population genetic analysis based on DNA sequences. However, based on the combined STR alleles and SNPs at all six MLST loci, 188 genotypes were found, and the combined genetic information was used for clonal correction for the following population genetic analyses.

Overall, in the MLST dataset, 19% of the total genetic variation was attributed to geographic separation among populations, and 81% was found within populations (PhiPT = 0.192; *p* = 0.006). The pairwise comparisons of the same dataset showed that among the 120 local population pairs, 29 pairs showed statistically significant differentiation (*p* < 0.05). The biggest differentiation was found between the Tibet and Hubei populations (PhiPT = 0.736; *p* = 0.004), followed by that between the Tibet and Guangxi populations (PhiPT = 0.647; *p* = 0.016) (Table S2). The Mantel test further showed that there was significant correlation between geographical distance and Nei genetic distance (*p* = 0.01) (Figure S2a). However, those from the STR dataset were quite different from the above-mentioned results revealed by MLST. AMOVA showed that more genetic variation (31% vs. 19% for the MLST dataset) was distributed among populations, and the remaining 69% genetic variations were found within populations in the STR dataset (PhiPT = 0.308; *p* = 0.001), indicating a higher level of genetic differentiation among populations by the new STR markers that that by sequence variation at the house-keeping gene based MLST. This pattern was similarly observed in the pairwise comparisons of genetic variance between populations, where the STR dataset showed that 95 out of 171 local population pairs were significantly differentiated (*p* < 0.05), which was more than those indicated by the MLST dataset. Based on STR markers, the Sichuan population was significantly different from all other geographic populations (*p* < 0.05) (Table S3). Moreover, the specific local population pairs with high genetic differentiation values were very different between the two datasets. The highest differentiation based on STR markers was between Inner Mongolia and Kanas Lake, Xinjiang populations (PhiPT = 0.994; *p* = 0.101, not significant), followed by that between Hubei and Sichuan populations (PhiPT = 0.869; *p* = 0.001) (Table S3). However, the correlation between geographical distance and Nei genetic distance indicated by the STR dataset was not significant (*p* = 0.24) (Figure S2b), which was not consistent with that of the MLST dataset.

AMOVA based on the combined dataset revealed that 72% of genetic variation was found within local populations, 28% of genetic differentiation was found among populations, and 132 out of 171 local population pairs showed statistically significant differentiation (*p* < 0.05); more of these pairs were found from populations from Southwest China (Sichuan, Guizhou, Tibet, and Yunnan including Dianchi, Heijing, Yimen, and Gejiu) and Hainan Island, as compared to those from other parts of China (Table S4). However, though there was a positive correlation between geographical distance and Nei genetic distance, such a correlation was statistically insignificant (*p* = 0.16) (Figure S2c).

**Figure 3:**
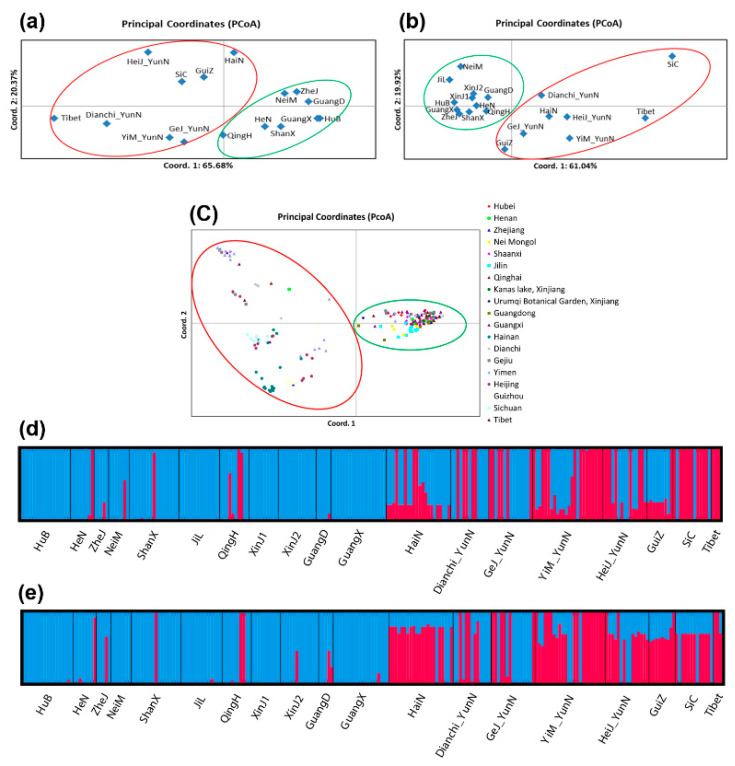
Genetic structuring results obtained from PCoA and STRUCTURE analyses. Analysis using MLST the dataset (**a**,**d**), the STR dataset (**b**,**e**), and the combined dataset (**c**).

Though significant genetic differentiations were found among many geographic populations, our analyses also showed evidence of gene flow among certain geographic populations. The existence of gene flow and genetic differentiation was further supported by results from the STRUCTURE analysis that showed that there were two genetic clusters (K = 2) in the Chinese population of *A. oligospora*, and most geographic populations contained alleles and genotypes of both genetic clusters. The population structure indicated by STRUCTURE software and PCoA was very similar, and both the MLST and STR datasets identified two distinct genetic clusters in all 239 *A. oligospora* isolates (Figure 3). Specifically, most samples from Southwestern China (Sichuan, Guizhou, Tibet, and Yunnan including Dianchi, Heijing, Yimen, and Gejiu) and Hainan Island formed one cluster, while those from other parts of China formed another cluster (Figure 3a–c). Populations in the former clade were found to have more frequent genetic exchanges than those in the second cluster (Figure 3d,e). However, the composition of two different genetic clusters indicated by the two kinds of molecular markers were quite different in some geographic populations. For example, STRs detected the existence of genetic elements of the second cluster in geographic populations from Hubei, Inner Mongolia, Jilin, Xinjiang, and Guangxi, while they were absent in the MLST dataset (Figure 3e). Interestingly, most genotypes containing elements of both genetic clusters (blue and red alleles in Figure 3d,e) were distributed in Southwestern China and Hainan. These genotypes likely represent hybrids of those two genetic clusters.

### 3.3. Phylogenetic Divergence

**Figure 4:**
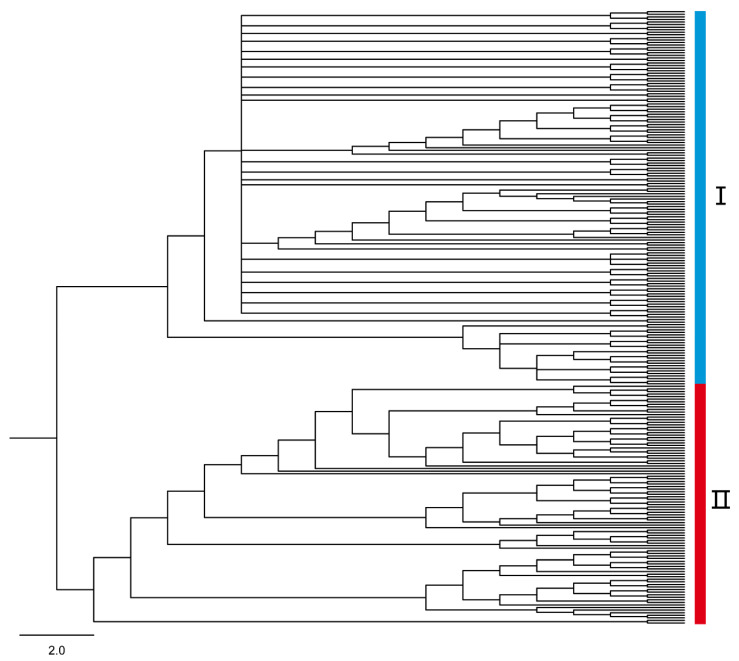
The topology structure of Bayesian phylogeny for the MLST SNPs. Note: all genotypes belonging to lineages I and II are indicated in the phylogenetic tree, the bars’ colors correspond to the genetic clusters indicated by STRUCTURE, and the strain numbers of each clade are listed in Table S5.

Phylogenetic analyses based on SNPs and MLST identified two distinct clades (Ⅰ and Ⅱ) within *A. oligospora* from 19 geographic populations in China (Figure 4, Figures S1 and S4; Table S5), consistent with the two genetic clusters revealed by the population structure analyses. Clades Ⅰ and Ⅱ of the MLST phylogeny included 38 and 21 multilocus sequence types, respectively. Most *A. oligospora* samples from different geographic populations (except for Guizhou) were widely distributed across Clade ⅠI. Interestingly, different geographical populations had different distribution patterns between the two clades: five geographic populations (Hubei, Jilin, Guangxi, and two populations from Xinjiang) were only found in Clade ⅠI, and samples from Tibet were only found in Clade I. Two populations (Hainan and Heijing from Yunnan) were widely distributed in both clades.

**Figure 5:**
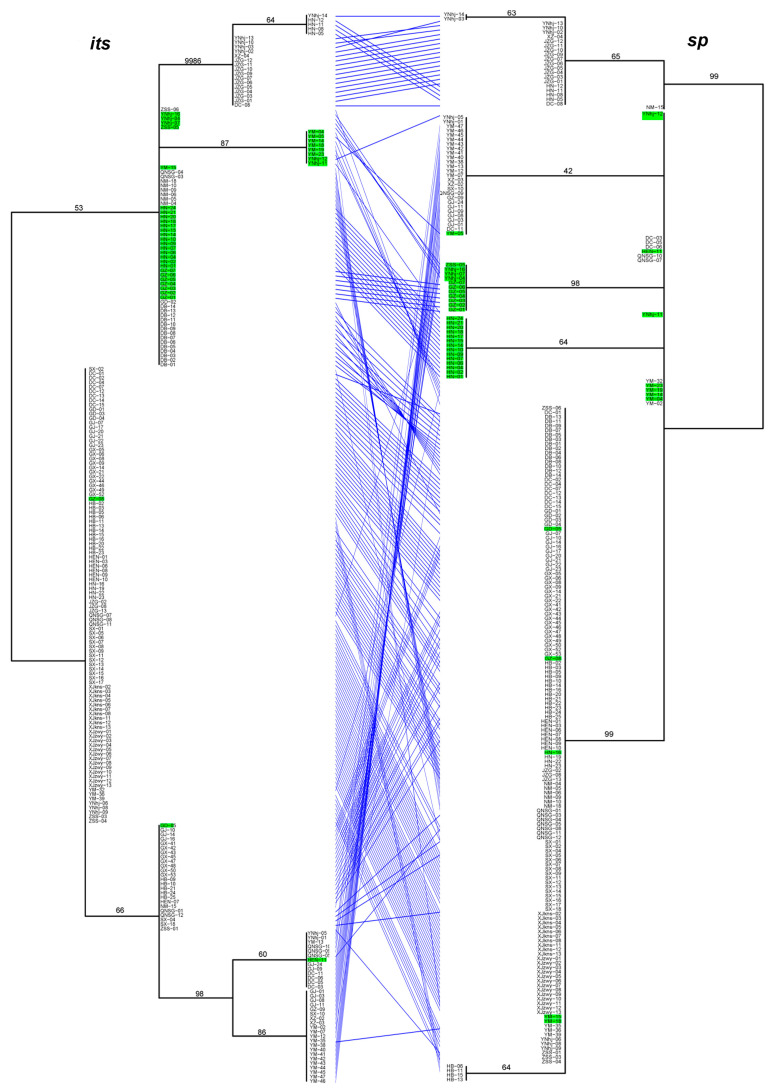
The tanglegram between *its* and *sp* phylogenies. Notes: Branch support values are indicated by numbers near branches, and putative hybrids (as suggested by STRUCTURE analysis) are colored in green in the tanglegram.

Significant differences in the relationships among strains among the analyzed gene fragments were identified based on the pairwise comparisons of topologies among all single gene phylogenetic trees (Figure 5 and Figure S3). The results indicated evidence of potential allele-sharing and/or hybridization between the genetic lineages of this important NTF. For example, strains containing both blue and red alleles in the STRUCTURE analysis (colored in green on trees) formed tight and independent clusters on trees of *mapk*, *rpb2* and *sp* fragments, suggesting they belonged to populations that are diverging from the other two clades. However, the same strains had mixed relationships with others based on sequences at *its*, *tub,* and *tef-1* loci. Together, these differences in phylogenetic relationships among strains by different gene fragments are consistent with recombination in nature.

### 3.4. Clonality and Recombination

A total of 197 *A. oligospora* strains were randomly selected, and their mating type (MAT) genes were successfully amplified. Among the 197 strains, 84 and 113 belonged to *mat1-1* and *mat1-2*, respectively, in a ratio of ~1:1.3, consistent with *A. oligospora* being a heterothallic fungus. Furthermore, the minimum spanning tree (MST) with the 197 strains based on the STR dataset showed that the *A. oligospora* strains containing *mat1-1* and *mat1-2* were both overall broadly distributed on the MST (Figure 2b). Indeed, some strains of different mating types shared the same STR genotype.

The rBarD and phylogenetic incompatibility tests for detecting recombination were conducted for (i) the total sample, (ii) samples from each geographical sample groups by both the STR and MLST datasets, and (iii) samples representing MLST genotypes in each of the phylogenetic clades (Table 4); the allelic sequences at each of the six MLST loci were treated as alleles in these tests. Though predominantly clonal population structures were detected for these sample sets, we found unambiguous evidences for recombination among the 20 STR loci (PrC = 0.01, *p* < 0.001; rBarD = 0.355, *p* < 0.001) and six MLST loci (PrC = 0, *p* = 1; rBarD = 0.6398, *p* < 0.001) in the total sample, including all 239 *A. oligospora* isolates. As indicated by the phylogenetic incompatibility test, variable levels of recombination were found within different sample groups. Interestingly, population groups from Southwestern China had the highest levels of phylogenetic incompatibility. It is worth noting that phylogenetic incompatibility was also identified in all samples where random recombination was rejected, indicating that low levels of recombination are common in samples of *A. oligospora* in China.

### 3.5. Phenotypical Characterization

#### 3.5.1. Conidial Morphology

For conidial morphology analyses, 38 strains of *A. oligospora*—including 30, 6, and 2 strains from the divergent Clades A, B, and C identified in our previous study [30], respectively—were selected to observe. Clades A and B respectively correspond to our Clades Ⅰ and Ⅱ here, and Clade C falls into the basal branch of Clade Ⅱ. After conidia sporulation on CMA, the length and width of 50 mature conidia of each strain were observed and measured, and the length–width (L/W) ratio was selected as the quantitative measure for the following statistical analyses.

**Figure 6:**
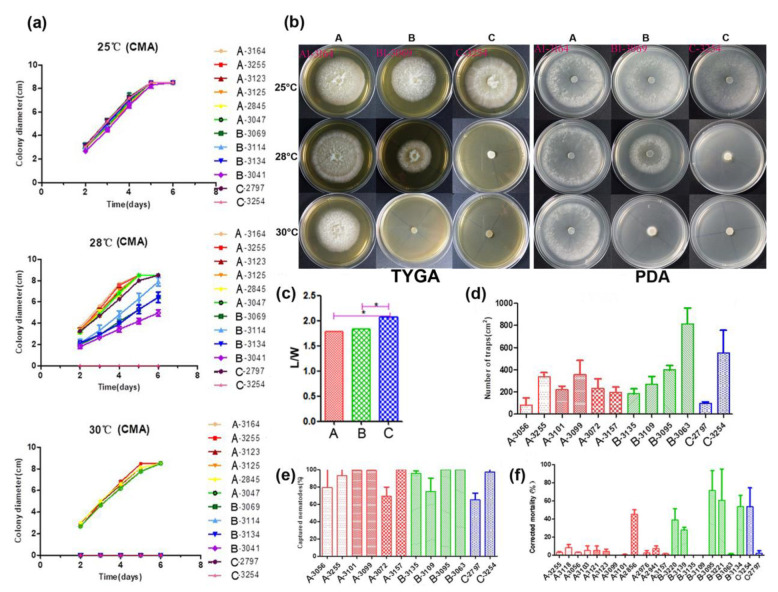
Phenotypical characterization of strains from the three main lineages. (**a**) The growth state of 12 representative *A. oligospora* strains incubation at 25, 28, and 30 °C on CMA. (**b**) Colony morphology of representative strains at 25, 28, and 30 °C on TYGA and PDA plates. (**c**) Pairwise comparisons of L/M among representative strains. (**d**) Comparison of trap formation, (**e**) captured nematodes, and (**f**) nematicidal activities of fermentation broth among representative strains. Notes: CMA: corn meal agar, TYGA: the tryptone, yeast extract, and glucose agar, PDA: potato dextrose agar, * significantly different.

First, we tested variance homogeneity among the data from three different phylogenetic clusters. A Levene’s *p* value of greater than 0.05 was obtained, suggesting there was no significant difference in the total variance among the three clusters. Second, an analysis of variance was conducted. The L/W ratio of Clade C strains was found to be significantly higher than those of Clades A and B by Duncan’s multiple comparison test (Figure 6c). The L/W ratios of strains from Clades A and B were not significantly different from each other. Together, our results indicated some morphological differentiation among the clades within *A. oligospora* strains from China.

#### 3.5.2. Mycelial Growth

A total of 12 strains representing three clades (six from Clade A, four from Clade B, and two from Clade C) from different geographic populations were selected to investigate their potential differences in mycelial growth rate and colony morphology on three different media at 25, 28, and 30 °C. The fungal colonies on the CMA medium were generally very loose and their aerial hyphae were sparse. By contrast, those on TYGA medium produced extremely dense mycelia and their aerial mycelia grew most robustly. The growth status of hyphae on the PDA medium was intermediate between CMA and TYGA (Figure 6b).

Overall, the optimal growth temperature of different *A. oligospora* strains was 25 °C. At 25 °C, all strains could grow normally and showed the fastest growth rate among the three tested temperatures. However, the growth rates of isolates from Clades B and C (except YMF1.02797 from Clade C) were slower than those of Clade A at 28 °C, and the aerial mycelia deviated from radial spread. In fact, isolates from Clades B and C were unable to grow at 30 °C (Figure 6a,b). Together, these results suggest clade-specific differences in colony morphology and mycelial growth, regardless of geographic origins.

#### 3.5.3. Conidial Yield, Trap Formation and Nematode-Trapping Bioassay

*A. oligospora*, which can capture nematodes by forming three-dimensional networks from specialized hyphae, is considered the most abundant nematode predator in temperate soils [11]. In addition, genetic differences have been reported to have effects on the response of *A. oligospora* to its prey, though robust correlation has not been observed [21]. Therefore, the conidial yield, trap formation, and nematode-trapping bioassays were further investigated to examine whether there were notable differences among nematicidal activities among the three phylogenetic clades identified in a previous study.

Overall, we found significant differences in conidial yield among strains, but the differences among phylogenetic clades were not statistically significant. Strains from each of the phylogenetic clades have variable abilities to produce conidia. For example, three strains from the three clades (with one representing each clade)—YMF1.03101 (Clade A), YMF1.03135 (Clade B) and YMF1.03254 (Clade C)—were found to have significantly higher conidial yields than all other tested strains from either the same or different clades.

The number of traps produced by different strains of *A. oligospora* was most distinguishing when exposed to *C. elegans* for 12 h. The highest number was produced by strain YMF1.03063 of Clade B at >47% more than others, while the numbers produced by YMF1.03056 of Clade A and YMF1.02797 of Clade C were the least, only accounting for about 20% of that produced by YMF1.03063. The number of trappers produced by other tested strains were similar (Figure 6d). Overall, there was no significant correlation between the various abilities of the trap formation and phylogenetic division of *A. oligospora* strains from China. Correspondingly, limited difference regarding nematode-trapping abilities was found among the three phylogenetic clusters (Figure 6e).

We further detected the pathogenicity of fermentation broth from different representative strains in the three phylogenetic clades. Forty-eight hours after the nematodes were added into the fermentation supernatants, the lethal effects of different *A. oligospora* strains on nematodes was significantly different. Generally, strains YMF1.02856 (Clade A), YMF1.03095 (Clade B), and YMF1.03254 (Clade C) had obvious nematicidal effect, and the corrected mortality caused by strain YMF1.03095 was as high as 71.5%. However, strains YMF1.03056 and YMF1.02797, which showed the distinguishing ability to form traps and capture nematodes on the solid media, produced fermentation supernatants with weak pathogenicity (Figure 6f). Overall, we found no significant difference among clades in terms of the nematicidal activity of their fermentation broth.

## 4. Discussion

### 4.1. Development of Novel STRs for NTF

For decades, molecular tools have acted as complementary techniques to differentiate species and examine phylogenetic relationships and systematics for NTF with traditional morphological methods [17,18,19,20,38]. Recently, MLST and RFLP have been used for ecological studies of nematophagous fungi [30,31], and Orbiliales-specific PCR primers were developed to directly detect NTF in environmental samples [52]. However, limited information is available on the patterns of genetic variations within and among geographic populations. In this study, we described a new panel of 20 STRs for the exact and high-resolution genotyping strains of *A. oligospora* from China. About 2/3 of these STR markers were identified as highly polymorphic, and abundant allelic and genotypic diversities were detected among isolates of *A. oligospora* from China, thus indicating the high discriminatory power of our newly developed STRs.

Traditionally, the sprinkle plates method is used to isolate and identify NTFs based on their colony morphology and type of nematode traps produced. However, it is often difficult to analyze such data to infer the relationships among strains and populations. For example, the sprinkle plate method may be biased toward detecting species of nematophagous fungi that are adapted for growth in culture and have good predacious ability [53]. Here, we showed that the STR markers were highly discriminatory for identifying alleles and genotypes, and they enabled the generation of reproducible research results that could be easily shared among investigators when analyzing NTFs. Furthermore, the interactions among fungi, nematodes, and other soil organisms in different soils and the population dynamics of NTFs could be better uncovered with these markers.

In this study, the overall genetic clustering and phylogenetic separation indicated by the two kinds of molecular marks (STR vs. MLST) were similar. However, differences concerning genetic differentiation and the level of genetic exchange (hybridization) were also found. Specifically, more genetic variation among populations and frequent gene exchanges were revealed by STRs, which was likely due to the faster evolutionary rate of STRs than MLST. Indeed, the STR markers allowed for the detection of hybridization in most geographic populations, suggesting the hybridization events likely occurred very recently. Similarly, STRs failed to identify a statistically significant positive correlation between genetic and geographic distances or to discriminate the ancient population divergence that the MLST revealed. In addition, though the STR markers showed overall higher population differentiation than the MLST markers, they also showed greater gene flow among many of the 19 geographical populations. Together, due to the high mutation rate, the STR markers allowed us to capture the recent, fine-scale population genetic events that the MLST markers failed to reveal. Future studies should focus on analyzing the potential factors contributing to the differences in population structures, as revealed by MLST and STR markers, including factors such as ecological niche adaptation and host nematode distributions and preferences.

### 4.2. Historical Population Differentiation

Our study identified two distinctly differentiated genetic clusters among *A. oligospora* strains in China, indicating historical population divergence in this species. One of the major genetic cluster was composed of samples from Southwestern China and Hainan Island. Geographic populations from this region had a large number of private genotypes and unique alleles. The widespread genetic diversity and uniqueness in these areas suggested historical geographic isolation and, likely, the accumulation of locally adapted genotypes within and among local populations. Factors such as landscape features (e.g., mountain ranges), climate, and habitats in different geographic populations could all impact local ecology, genetic drift, selection, and adaptation. Specifically, Southwest China has a large number of mountain ranges and river systems that create a diversity of ecological niches, making it one of the most important biodiversity hot spots on earth [54]. Indeed, the high species diversity and endemism of fungal species and genotypes have been frequently revealed in this area [55]. For example, our recent population genetic investigations revealed multiple independent origins of the *Aspergillus fumigatus* pathogenic hyphomycetes in Yunnan, and they were significantly different from those in other parts of the world [56,57]. Furthermore, Hainan is the only island population with a tropical monsoon oceanic climate, and the geographic isolation between Hainan and the rest of China may result in the accumulation of unique genetic diversity. Together, the geographic and climatic factors likely contribute to the observed unique genetic diversities within individual local populations.

### 4.3. Recent Hybridization and Recombination

Our analyses revealed evidence for genetic exchanges among the strains and populations in Southwestern China and Hainan Island. As indicated by the population structure and gene genealogical analyses, there is extensive evidence for allele-sharing among lineages of *A. oligospora*, most likely due to hybridization. In addition to inter-lineage hybridization, we also found unambiguous evidence for recombination within individual lineages, though most of the samples showed evidence of non-random recombination. The frequent sharing of alleles but limited or no overlap in multilocus genotypes were further consistent with frequent recombination in nature.

The similar frequencies of *mat1-1* and *mat1-2* idiomorphs in our samples were also consistent with sexual reproduction and heterothallic lifecycle of *A. oligospora* in nature. Indeed, the widespread distributions of both *mat1-1* and *mat1-2* idiomorphs across geographic populations made mating between strains with different genetic elements possible. The finding that a couple of strains with different mating types shared the same STR genotype is also a strong piece of evidence for their recombinant origins. Our results here complement those in an earlier study that showed *A. oligospora* populations from stressful environments to be in greater linkage equilibrium than those from other environments in the same geographic areas [31].

In addition to extensive allelic and multilocus genotypic sharing (hybrids) in our samples, our other analyses such as gene genealogical comparison and STRUCTURE also suggested frequent hybridizations, with different strains showing variable proportions of genetic elements from the two genetic clusters. Similar population structures have been found in several other fungal populations in Southern China, such as those of the opportunistic human fungal pathogens *Aspergillus fumigatus* in Yunnan [56] and *Candida tropicalis* in Hainan Island [58], the plant pathogen *Fusarium oxysporum* in South-Central China [59], and the wild edible mushroom *Russula virescens* Ally from Southwestern China [60]. Our results were consistent with the idea of gene flow being a major force shaping the *A. oligospora* population from China. As indicated in our previous studies, the long-distance dispersal of asexual spores by natural forces, anthropogenic activities, the transport of agricultural and forestry products, and animal (soil nematode and insects) vectors all could have contributed to the spread of genetic elements of *A. oligospora* to different ecological and geographic niches [30]. With increasing anthropogenic influences such as human travel and the transport of good and services among regions, gene flow among geographic populations of *A. oligospora* will likely increase, which could further facilitate hybridization and recombination, potentially accelerating the distribution of adaptive alleles originating in one area to broader geographic reaches [61].

### 4.4. Intraspecies Diversification

The Fungal kingdom is among the most diverse and specious groups of eukaryotes. At present, we are still far from knowing the full extent of fungal diversity. One major finding based on DNA sequences over the last two decades has been the existence of multiple genetically divergent cryptic species within many previously defined species [61,62,63,64,65]. Within NTF, morphology-based classifications of strains and species have also likely underestimated the true magnitude of species diversity in this group of fungi. Though the globally isolated 22 isolates of a promising biocontrol agent *Duddingtonia flagrans* have a single recent common ancestor with limited genetic differentiation among geographic populations [66], several species within the NTF genera *Arthrobotrys* and *Monacrosporium* have been found to contain significant genetic variation and cryptic species among morphologically similar isolates [67].

Over the last two decades, the phylogenetic species concept (PSC) has been frequently used for identifying fungal species [64]. According to the PSC, species are diagnosed as a cluster of individuals that are sufficiently differentiated from other clusters, as revealed by DNA sequences. Given the presence of genetically differentiated clusters of strains in the phylogenetic tree, we sought to further determine whether the observed genetic divergence within *A. oligospora* was associated with phenotypic characters such as morphology and nematode-trapping abilities. As indicated by shape of conidia (L/W), the morphological differentiation of *A. oligospora* strains was found to be associated with phylogenetic lineage differentiations. However, we also observed large variations among strains within each of the lineages, a result consistent with significant observed genetic variations within individual lineages of this species. The observed variations suggest in field applications as biocontrol agents against plant-parasitic nematodes, a mixture of genotypes from the locally sourced strains might be more effective than a single strain.

The genetic clusters and phylogenetic lineages identified here represent speciating populations within *A. oligospora*. At the same time, the identification of hybrids in most geographic populations suggests that strains in different clusters/lineages are sexually compatible. Indeed, the comparable distributions of *mat1-1* and *mat1-2* genes suggest that sexual mating is likely common in natural populations of this species. Analyses of laboratory crosses between strains from the same and different lineages could help reveal the underlying genetic mechanisms responsible for the observed phenotypic variations. Indeed, we are in the process of identifying strains of opposite mating types and conducting laboratory crosses for strains between lineages to examine their cross-fertility and the extent of potential reproductive isolation. Species and cryptic species have been defined based on many different criteria [61], including geographical origin [68], ecological adaptation [69,70], and host specialization [71]. Host preference has been found in pairs of tightly interacting hosts and pathogens due to shared common evolutionary histories. However, in facultative pathogens, the selective pressure exerted by both hosts and habitats could be equally important [72,73]. At present, the extent of nematode-host specificity and how such specificity may be related to phenotypic diversification and ecological niche adaptation remain largely unknown.

In selecting strains for field applications as biocontrol agents against PPNs, a common approach is to select strains with the strongest nematode-trapping ability, as demonstrated in laboratory settings. However, we believe many other factors, including the specificity of NTF–PPN interactions and the growth and reproductive abilities of the selected strains in field conditions, should be considered. The large number of strains and genotypes identified here for *A. oligospora* from China will allow us to test the importance of some of these factors for future biocontrol applications.

## Figures and Tables

**Table 1:** STR allele distributions within and among 19 geographic populations of *A. oligospora* for each of the 20 STR loci.

Population	No. Isolates	No. Alleles in Each Locus (No. Private Alleles)																				
		A3	A5	A17	A25	A51	A74	A80	A83	A87	A101	A103	A126	A149	A154	A156	A160	A177	A187	A191	A192	Total
Hubei (HuB)	17	1	1	3	2	1	2	1	2	1	2	2	2	2 (1)	2	2	3 (1)	3	2	3	1	38 (2)
Henan (HeN)	8	2	1	2 (1)	1	1	3	2	2	2	3	3	2	2	2	3	2	2	3	5	2	45 (1)
Zhejiang (ZheJ)	5	1	2	3	1	2 (1)	3	2	2	1	4	2	2	1	1	3	1	4	2	3	2	42 (1)
Nei Mongol (NeiM)	7	1	1	1	1	1	1	1	1	1	1	2	1	1	1	2	1	1	2	1	1	23
Shaanxi (ShanX)	17	2	1	3	2	2	4	2	4	2	5	5	4 (1)	2	3	3	3	3	3	4	2	59 (1)
Jilin (JiL)	14	1	1	2	1	1	3	1	2	1	3	2	2	1	3 (1)	2	1	1	2	3 (1)	1	34 (2)
Qinghai (QingH)	10	2	2	3	3	2	4	3	2	2	5	5	3	2	3	3	3	5	3	5	3	63
Kanas lake, Xinjiang (XinJ1)	10	1	1	1	1	1	1	1	1	1	1	2	1	1	1	1	1	1	1	1	1	21
Urumqi Botanical Garden, Xinjiang (XinJ2)	13	1	1	2	2	2	1	2	2	1	2	2	2	1	2 (1)	2	2	1	2	1	1	32 (1)
Guangdong (GuangD)	5	1	1	4	1	2	2	2	2	2	5	2	3	2	1	3 (1)	2	2	2	4	2	45 (1)
Guangxi (GuangX)	19	1	1	1	1	2	3	1	3	2 (1)	5 (2)	3	2	2	2	2	2 (1)	3	2	4 (1)	2 (1)	44 (6)
Hainan (HaiN)	22	1	3	4	5	3	3	3	5	4	4	4	4 (1)	6 (2)	2	3	2	4	3	5 (2)	3	71 (5)
Dianchi lake, Yunnan (Dianchi_YunN)	13	3	3	2	4	2	3	4	4 (2)	4 (1)	4	5	6	4	2	3	3	4	3	4 (1)	4	71 (4)
Gejiu, Yunnan (GeJ_YunN)	15	3	2	3	3	4	3	3	3	3	5	5 (1)	2	3	3	4 (1)	3 (1)	4	3 (1)	5	3 (1)	67 (5)
Yimen, Yunnan (YiM_YunN)	24	3	2	4	3	3	4	6 (2)	5 (1)	3	5 (1)	3	4	4	3	4	4 (2)	6 (2)	3	7 (1)	3	79 (9)
Heijing, Yunnan (HeiJ_YunN)	15	2	5	2	3	3	5	3	5	4	8 (1)	4	5	2	4 (1)	4	5	5	6	8 (1)	5	88 (3)
Guizhou (GuiZ)	9	2	2	3	1	3 (1)	2	2	3	2	3 (1)	3	3	2	2	3	2	2	3	2	2	47 (2)
Sichuan (SiC)	13	1	2	2	2	2	3	2	3	2	3	3 (1)	2	3	1	2	2	3	2	2	2	44 (1)
Tibet	3	2	2	2	2	3	2	2	2	3 (1)	1	2	2	2 (1)	2	2	3	1	3 (2)	1	2	41 (4)
Total	239	3	5	7	5	9	10	7	10	8	16	13	10	11	6	8	11	12	9	21	7	188 (48)

**Table 2:** Characterization of 20 STR loci that were newly developed in this study.

Locus	Forward Primer Sequence	Reverse Primer Sequence	Repeat Unit	PIC	Allele Frequency	Availability	Gene Diversity
A3	FAM-GGAGTGGAAGTTAGATTGGAG	GGGGAACTAATTTACTTGCAT	GTG	0.244	0.849	0.941	0.265
A5	HEX-CGATGAAGACGTGAGTTAGTT	TGTCTGTCACCACCTTTAATC	GATG	0.333	0.795	0.958	0.353
A17	TET-CTCTGCTGAGACGTTAATGAT	GTAAATCGTACCCAAGAGGTT	TGG	0.534	0.563	0.967	0.593
A25	FAM-GGGCATACCTCTCTTCTCTTA	GGATTGCTAGGTATGGTCTTT	AGT	0.347	0.785	0.954	0.367
A51	HEX-ATTAACAATGGTCCGAAACTT	GACAAGGAGAAAGCCATTAGT	AAGC	0.465	0.702	0.941	0.486
A74	TET-GATCGATTCTCGCTTAAAGAC	TCCTGCTCCACTATACTCTCA	TGC	0.790	0.286	0.979	0.814
A80	FAM-GGGACATCGACAATATGTAAG	GAGCTCTGCTTTGAGACATAA	CTG	0.552	0.603	0.937	0.588
A83	HEX-GAATCTTTCGGTTTAATGGTT	GGGAATGGTGGTATCATAGTA	CTTT	0.644	0.532	0.975	0.671
A87	TET-GGAGAAACATCAATCAATCAA	CTGAGAGGAACCAAGATGTC	AGCA	0.508	0.651	0.958	0.540
A101	FAM-ACAACATCAACTACCATCCAC	GGCTATTGGAAGAAGGATAAG	CTC	0.811	0.330	0.925	0.828
A103	HEX-TCACTGCACTATCTCCAATCT	ACACGACATCGAAACATACTT	TGTA	0.734	0.329	0.979	0.765
A126	TET-GCCAGGTGGTTAGGAGTATAA	TATTTGAACCACCATAACGTC	AAAG	0.697	0.420	0.967	0.733
A149	FAM-AAAGAATGTGTGTCATCGAAT	TTCATCCTAGTTCCGTCAGTA	CA	0.458	0.714	0.908	0.474
A154	HEX-TAATCTGAATGGTTGGTTGTT	CATGAAGGACTGTCAACTAGC	GCTA	0.475	0.640	0.954	0.528
A156	TET-ATGTTTAATTTCCCTCCAAAC	TTCTCTCAACTCGCAATTCT	TGA	0.682	0.475	0.933	0.712
A160	FAM-GAACATGCACGTGTGAGATA	GACTCCATACGAGACCATACA	AGTC	0.430	0.726	0.933	0.452
A177	HEX-GTTCGAGGGATAGTAGTGGTT	CCAACGCATATCTTTTACCTA	AG	0.712	0.358	0.912	0.747
A187	TET-GTCCAAGTTTGTCCAGTACAC	ATCGTGGAGAATATACCGAAT	CCT	0.628	0.491	0.937	0.672
A191	FAM-AACACATCTCATTCATCCATC	ACCTGACATTTGACAGTTGAC	CAC	0.858	0.225	0.950	0.870
A192	HEX-CCTAATACCCAACCGAATAAC	AAACAGGTGTAACTGGGTTCT	CTC	0.541	0.610	0.933	0.579

Notes: PIC: polymorphism information content.

**Table 3:** Distribution and diversity of MLST genotypes from 19 geographic populations of *A. oligospora* in China.

Population	No. of Isolates	MLST Genotype (No. of Isolates in Each Genotype)	Private MLST Genotype	Genotypic Diversity
HuB	17	1 (3); 2 (1); 4 (1); 21 (1); 23 (4); 28 (1); 29 (1); 30 (3); 36 (2)	4; 21; 28; 29; 30	0.904
HeN	8	1 (1); 23 (4); 24 (1); 25 (1); 50 (1)	25; 50	0.786
ZheJ	5	1 (1); 7 (1); 16 (1); 23 (2)		0.900
NeiM	7	5 (1); 6 (2); 7 (4)	5; 6	0.667
ShanX	17	1 (1); 2 (1); 19 (1); 20 (1); 23 (10); 24 (2); 57 (1)	19; 20; 57	0.662
JiL	14	7 (14)		0
QingH	10	1 (1); 2 (1); 7 (2); 23 (2); 31 (1); 47 (1); 51 (1); 52 (1)	31; 47; 51; 52	0.956
XinJ1	10	23 (10)		0
XinJ2	13	23 (13)		0
GuangD	5	3 (1); 7 (1); 23 (3)	3	0.700
GuangX	19	1 (7); 2 (1); 22 (1); 23 (4); 24 (6)		0.754
HaiN	22	9 (1); 10 (1); 11 (10); 12 (1); 13 (1); 22 (1); 23 (2); 26 (1); 41 (4)	9; 10; 11; 12; 13; 26; 41	0.775
Dianchi_YunN	13	23 (8); 45 (1); 48 (1); 49 (2); 53 (1)	48; 49	0.628
GeJ_YunN	15	1 (3); 23 (6); 53 (2); 58 (4)		0.286
YiM_YunN	24	8 (1); 33 (1); 34 (4); 35 (1); 37 (1); 38 (1); 39 (1); 53 (1); 54 (1); 55 (1); 56 (1); 58 (9); 59 (1)	8; 33; 35; 37; 38; 39; 54; 55; 56; 59	0.848
HeiJ_YunN	15	14 (1); 16 (1); 18 (1); 24 (1); 32 (1); 34 (1); 36 (2); 40 (1); 42 (1); 44 (1); 45 (1); 46 (1); 53 (2)	14; 18; 32; 40; 42; 44; 46	0.981
GuiZ	9	15 (1); 16 (5); 17 (1); 27 (1); 58 (1)	15; 17; 27	0.722
SiC	13	23 (3); 45 (10)		0.385
Tibet	3	43 (1); 58 (2)	43	0.667

Notes: MLST: multilocus sequence typing.

**Table 4:** Results of multilocus linkage disequilibrium analyses for different sample groups of *A. oligospora* from China.

Sample Groups	Sample Size	Phylogenetic Compatibility (*p* Value)		rBarD (*p* Value)	
		MLST	STR	MLST	STR
Total	239	0 (1)	0.01 (<0.001)	0.6398 (<0.001)	0.355 (<0.001)
Central	25	0.8667 (0.447)	0.7 (0.038)	0.1077 (0.009)	0.1331 (<0.001)
East	5	1 (1)	0.9947 (0.096)	0.6805 (0.001)	0.3039 (<0.001)
North	24	0.9333 (1)	0.8 (<0.001)	0.2784 (<0.001)	0.2772 (<0.001)
Northeast	14	1 (1)	0.9211 (0.009)	-nan * (<0.001)	0.115 (<0.001)
Northwest	33	0.8 (0.889)	0.8632 (<0.001)	0.576 (<0.001)	0.3988 (<0.001)
South	46	0.4667 (<0.001)	0.2526 (<0.001)	0.6867 (<0.001)	0.288 (<0.001)
Southwest	92	0.2667 (<0.001)	0.0579 (<0.001)	0.661 (<0.001)	0.3874 (<0.001)
Clade I	151	0.1333 (<0.001)	0.021 (<0.001)	0.5042 (<0.001)	0.2019 (<0.001)
Clade II	46	0.5333 (<0.001)	0.3052 (<0.001)	0.6379 (<0.001)	0.4439 (<0.001)

Notes: Geographic populations included in each of the major regions in China: Central: Hubei, Henan; East: Zhejiang, Shandong; North: Hebei, Nei Mongol; Northeast: Jilin; Northwest: Shaanxi, Qinghai, Xinjiang; South: Guangdong, Guangxi, Hainan; Southwest: Sichuan, Guizhou, Yunnan, Tibet. * All strains in this population have the same haplotypes in six gene fragments. MLST: multilocus sequence typing; STR: short tandem repeat; rBarD: standardized index of association.

## Data Availability

The MLST data presented in this study can be found in online repositories. The names of the repository/repositories and accession numbers can be found below: https://www.ncbi.nlm.nih.gov/genbank/ (accessed on 1 August 2021), *its*: MZ571947~MZ572185, *mapk*: MZ616125~MZ616363, *rpb2*: MZ596984~ MZ597222, *sp*: MZ596745~MZ596983, *tef-1*: MZ606385~MZ606623, *tub*: MZ597223~MZ597461.

## Supplementary Materials

The following are available online at https://www.mdpi.com/article/10.3390/microorganisms9091919/s1. Figure S1: Genetic clustering of 239 *A. oligospora* isolates from China based on 20 STR loci by Nei’s distance. Notes: Scale bar indicates percentage identity. Figure S2: Mantel tests of the relationships among genetic differentiation (Nei values) and geographical distance. (**a**) MLST dataset (*p* = 0.01), (**b**) STR dataset (*p* = 0.24), and (**c**) combined dataset (*p* = 0.4). Figure S3: 14 Pairwise tanglegrams between single gene phylogenies. Notes: Hybrids indicated by structure analysis are colored in green on the tanglegram. Figure S4: Bayesian phylogeny for the MLST SNPs. Table S1: Length and polymorphism information of six sequenced DNA fragments. Table S2: Pairwise differentiations of *A. oligospora* isolates from 16 geographic populations from China based on MLST dataset. Notes: Values in the bottom left represent PhiPT values, while those in the top right represent *p* values. Table S3: Pairwise differentiations between 19 geographic populations of *A. oligospora* in China based on the STR dataset. Notes: Values in the bottom left represent PhiPT values, while those in the top right represent *p* values. Table S4: Pairwise differentiations between 19 geographic populations of *A. oligospora* in China based on a combined MLST and STR dataset. Notes: Values in the bottom left represent PhiPT values, while those in the top right represent *p* values, Table S5 List of samples used in Figure 4.
