# Supplementary material for: Historical Differentiation and Recent Hybridization in Natural Populations of the Nematode-Trapping Fungus Arthrobotrys oligospora in China
Source: Microorganisms. 2021 Sep 9;9(9):1919. doi: 10.3390/microorganisms9091919 (PMC8465350; doi:10.3390/microorganisms9091919)
Supplement: Supplementary file 1 [file microorganisms-09-01919-s001.zip › Figure S1 Genetic clustering of 239 A. oligospora.pdf]

STR (<All Characters>)

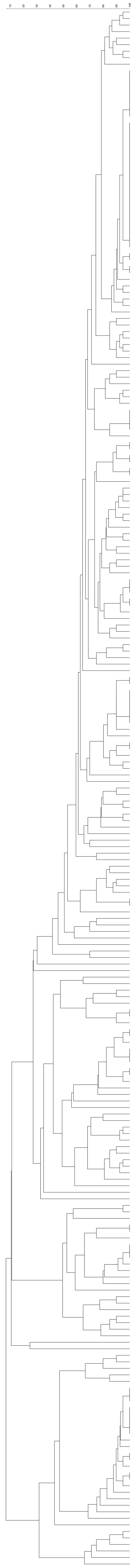

STR

位点3	位点5	位点17	位点25	位点51	位点74	位点80	位点83	位点87	位点101	位点103	位点126	位点149	位点154	位点156	位点160	位点177	位点187	位点191	位点192	Sample	site
136	152	155	135	144	161	135	142	160	156	142	194	132	164	137	145	139	139	179	157	GD01	Guangdong
136	152	155	135	144	161	135	142	160	156	142	195	132	164	137	145	139	139	179	157	HEN01	Henan
136	152	155	135	144	161	135	142	160	156	142	195	132	164	137	145	143	139	179	157	QNSG11	Qinghai
136	152	155	135	144	161	135	142	160	156	141	195	132	164	137	145	139	139	176	157	YNhj06	Heijing_Yunnan
136	152	155	135	144	161	135	142	160	179	142	195	132	164	137	145	143	139	176	157	GD03	Guangdong
136	152	155	135	144	161	135	142	160	156	143	195	132	164	137	145	143	139	176	157	HB22__F02.fsa	Hubei
136	152	155	135	144	164	135	142	160	156	142	194	132	164	137	145	143	139	176	157	HEN08	Henan
136	152	155	135	144	164	135	142	160	156	142	195	132	164	137	145	143	139	176	157	HEN10	Henan
136	152	155	135	144	161	135	143	160	156	143	194	132	164	137	145	143	139	176	157	HB16__C02.fsa	Hubei
136	152	155	135	144	161	135	142	160	156	142	195	132	164	138	145	143	140	176	157	XJkns04	Xinjiankns
136	152	155	135	144	161	135	142	160	156	142	195	132	164	138	145	143	140	176	157	XJzwy07	Xinjiangzwy
136	152	155	135	144	161	135	142	160	156	142	195	132	164	138	145	143	140	176	157	XJzwy08	Xinjiangzwy
136	152	155	135	144	161	135	142	160	156	142	195	132	164	138	145	143	140	176	157	XJzwy11	Xinjiangzwy
136	152	155	135	144	161	135	142	160	156	142	195	132	164	138	145	143	140	176	157	XJzwy12	Xinjiangzwy
136	152	155	135	144	161	135	142	160	156	142	195	132	164	138	145	143	140	176	157	XJzwy13	Xinjiangzwy
136	152	155	135	144	161	135	142	160	156	142	195	132	164	138	145	143	140	176	157	DL15__C07.fsa	Dianchi_Yunnan
136	152	155	135	144	161	135	142	160	156	142	195	132	164	138	145	143	140	176	157	XJzwy10	Xinjiangzwy
136	152	155	135	144	161	135	142	160	156	143	195	132	164	138	145	143	140	176	157	JZG02	Sichuan
136	152	155	135	144	161	135	142	160	156	143	195	132	164	138	145	143	140	176	157	JZG13	Sichuan
136	152	155	135	144	161	135	142	160	156	143	195	132	164	138	145	143	140	176	157	XJkns02	Xinjiankns
136	152	155	135	144	161	135	142	160	156	143	195	132	164	138	145	143	140	176	157	XJkns03	Xinjiankns
136	152	155	135	144	161	135	142	160	156	143	195	132	164	138	145	143	140	176	157	XJkns05	Xinjiankns
136	152	155	135	144	161	135	142	160	156	143	195	132	164	138	145	143	140	176	157	XJkns06	Xinjiankns
136	152	155	135	144	161	135	142	160	156	143	195	132	164	138	145	143	140	176	157	XJkns07	Xinjiankns
136	152	155	135	144	161	135	142	160	156	143	195	132	164	138	145	143	140	176	157	XJkns08	Xinjiankns
136	152	155	135	144	161	135	142	160	156	143	195	132	164	138	145	143	140	176	157	XJkns11	Xinjiankns
136	152	155	135	144	161	135	142	160	156	143	195	132	164	138	145	143	140	176	157	XJkns12	Xinjiankns
136	152	155	135	144	161	135	142	160	156	143	195	132	164	138	145	143	140	176	157	XJzwy04	Xinjiangzwy
136	152	155	135	144	161	135	142	160	156	143	195	132	164	138	145	143	140	176	157	XJzwy03	Xinjiangzwy
136	152	155	135	144	161	135	142	160	156	143	195	132	164	138	145	143	140	176	157	HB3__B01.fsa	Hubei
136	152	155	135	144	161	135	142	160	156	143	195	132	164	138	145	143	140	176	157	HB5__C01.fsa	Hubei
136	152	155	135	144	161	135	142	160	156	143	195	132	164	138	145	143	140	176	157	DL1__G05.fsa	Dianchi_Yunnan
136	152	155	135	144	161	135	142	160	156	143	195	132	164	138	145	143	140	176	157	DL12__H06.fsa	Dianchi_Yunnan
136	152	155	135	144	161	135	142	160	156	143	195	132	164	138	145	143	140	176	157	DL13__A07.fsa	Dianchi_Yunnan
136	152	155	135	144	161	135	142	160	156	143	195	132	164	138	145	143	140	176	157	DL2__H05.fsa	Dianchi_Yunnan
136	152	155	135	144	161	135	142	160	156	143	195	132	164	138	145	143	140	176	157	JZG08	Sichuan
136	152	155	135	144	161	135	142	160	156	143	195	132	164	138	145	143	140	176	157	XJzwy05	Xinjiangzwy
136	152	155	135	144	161	135	142	160	156	142	195	132	164	138	145	143	140	173	157	SX14__G11.fsa	Shanxi
136	152	155	135	144	161	135	142	160	156	142	195	132	164	138	145	143	140	173	157	SX9__B11.fsa	Shanxi
136	152	155	135	144	161	135	142	160	156	143	195	132	164	138	145	143	140	173	157	SX1__C10.fsa	Shanxi
136	152	155	135	144	161	135	142	160	156	143	195	132	164	138	145	143	140	173	157	SX2__D10.fsa	Shanxi
136	152	159	135	144	161	135	142	160	156	142	195	132	164	138	145	143	140	176	157	HEN07	Henan
136	152	155	135	144	161	135	142	160	156	143	195	132	164	138	145	139	140	176	157	QNSG8	Qinghai
136	152	155	135	144	161	135	142	160	156	143	194	132	164	138	145	139	140	176	157	HB2__A01.fsa	Hubei
136	152	155	135	144	161	135	142	160	179	142	194	132	164	138	145	143	140	176	157	ZSS04	Zhejiang
136	152	155	135	144	161	135	142	160	156	142	194	132	164	138	145	143	140	176	157	HEN09	Henan
136	152	155	135	144	161	135	141	160	156	142	195	132	164	138	145	143	140	173	157	SX13__F11.fsa	Shanxi
136	152	155	135	0	161	0	142	160	156	142	195	132	164	138	145	143	140	176	157	XJkns13	Xinjiankns
136	152	155	135	0	161	0	142	160	156	143	195	132	164	138	145	143	140	173	157	SX15__H11.fsa	Shanxi
136	152	155	135	0	161	0	142	0	156	143	0	132	164	138	145	143	140	173	157	SX16__A12.fsa	Shanxi
136	152	155	135	0	161	0	142	0	156	143	0	132	164	138	145	143	140	176	157	HB14__A02.fsa	Hubei
136	152	155	135	0	161	0	142	0	156	142	0	132	164	138	145	143	140	176	157	XJzwy02	Xinjiangzwy
136	152	155	135	0	161	0	142	0	156	142	0	132	164	138	145	143	140	173	157	SX8__A11.fsa	Shanxi
136	152	155	135	0	161	0	142	0	156	143	188	132	164	138	145	143	140	176	157	DL7__E06.fsa	Dianchi_Yunnan
0	0	0	0	144	161	135	142	160	156	143	195	132	164	138	145	143	140	176	157	XJzwy09	Xinjiangzwy
136	152	155	135	144	164	135	142	160	173	142	194	132	164	137	145	139	139	182	157	HEN03	Henan
136	152	155	135	144	164	135	142	160	173	141	194	132	164	137	145	139	139	179	157	YNhj09	Heijing_Yunnan
136	152	155	135	144	164	135	142	160	168	143	194	132	164	137	145	139	139	185	157	GX6__A05.fsa	Guangxi
136	152	155	135	144	161	135	142	160	156	143	194	132	164	137	145	139	139	179	157	QNSG1	Qinghai
136	152	155	135	144	161	135	142	160	180	143	194	132	164	137	145	139	139	179	157	GX47__E06.fsa	Guangxi
136	152	155	135	144	161	135	142	161	180	143	194	132	164	137	145	139	139	179	157	GX48__F06.fsa	Guangxi
136	152	155	135	144	161	135	141	160	179	143	194	132	164	138	145	139	140	179	157	GX41__G05.fsa	Guangxi
136	152	155	135	144	161	135	141	160	179	143	194	132	164	138	145	139	140	179	157	GX43__A06.fsa	Guangxi
136	152	155	135	144	161	135	141	160	179	143	194	132	164	138	145	139	140	179	157	GX50__H06.fsa	Guangxi
136	152	155	135	144	161	135	141	160	179	143	194	132	164	138	145	139	140	179	157	GX53__B07.fsa	Guangxi
136	152	155	135	144	161	135	142	160	168	143	194	132	164	138	145	143	140	179	157	GX42__H05.fsa	Guangxi
136	152	152	135	144	164	135	142	160	156	143	194	132	165	138	145	143	140	179	157	HB11__G01.fsa	Hubei
136	152	152	135	144	164	135	142	160	156	143	194	132	165	138	145	143	140	179	157	HB6__D01.fsa	Hubei
136	152	152	135	144	164	135	142	160	156	143	195	132	165	138	145	141	140	179	157	QNSG4	Qinghai
136	152	152	135	144	164	135	142	160	156	143	195	132	165	138	145	141	140	179	157	QNSG3	Qinghai
136	152	152	135	144	164	135	142	160	156	143	195	132	165	138	145	139	140	187	157	DB14__G04.fsa	Jilin
136	152	152	135	144	164	135	142	160	156	143	195	132	165	138	145	139	140	187	157	DB8__A04.fsa	Jilin
136	152	155	135	144	164	135	141	160	156	143	195	132	165	138	150	143	140	167	157	GX22__F05.fsa	Guangxi
136	152	155	135	144	164	135	142	160	173	142	194	132	165	138	145	150	140	179	157	HN19__C04.fsa	Hainan
136	152	155	135	144	164	135	142	160	174	142	194	132	165	138	145	150	140	179	157	HN22__F04.fsa	Hainan
136	152	155	135	144	164	135	142	160	168	142	194	132	165	138	145	150	140	179	157	HB9__E01.fsa	Hubei
136	152	155	135	144	164	135	143	160	174	142	194	132	165	138	145	150	140	179	157	HN23__G04.fsa	Hainan
136	152	155	135	144	164	135	142	160	156	142	194	132	165	138	145	143	140	179	157	GX5__H04.fsa	Guangxi
136	152	155	135	144	164	135	142	160	168	142	194	132	165	138	145	143	140	179	157	SX4__E10.fsa	Shanxi
136	152	155	135	144	164	135	142	160	168	143	195	132	165	138	145	150	140	179	157	QNSG7	Qinghai
136	152	155	135	144	163	135	142	160	168	142	194	132	165	138	145	139	140	185	157	SX12__E11.fsa	Shanxi
136	152	155	135	144	164	135	142	160	168	142	194	132	165	138	145	139	140	185	157	SX17__B12.fsa	Shanxi
136	152	155	135	144	164	135	142	160	156	142	194	132	164	138	145	139	140	179	157	ZSS06	Zhejiang
136	152	155	135	144	164	135	142	160	168	141	194	132	164	138	145	139	140	179	157	YNhj08	Heijing_Yunnan
136	152	155	135	144	164	135	141	160	168	143	194	132	164	138	145	139	140	167	157	GX44__B06.fsa	Guangxi
136	152	155	135	144	164	135	141	160	168	142	194	132	165	138	145	139	140	167	157	GX46__D06.fsa	Guangxi
136	152	155	135	144	164	135	142	160	0	143	194	132	165	138	145	139	140	167	157	GX21__E05.fsa	Guangxi
136	152	155	135	144	164	135	142	160	174	144	195	132	165	138	145	139	140	179	157	GJ20__F01.fsa	Gejiu_Yunnan
136	152	155	135	144	164	135	142	160	174	144	195	132	165	138	145	139	140	179	157	GJ21__G01.fsa	Gejiu_Yunnan
136	152	155	135	144	164	135	142	160	174	144	195	132	165	138	145	139	140	179	157	GJ22__H01.fsa	Gejiu_Yunnan
136	152	155	135	144	164	135	142	160	173	144	195	132	165	138	145	139	140	179	157	SX6__G10.fsa	Shanxi
136	152	155	135	144	164	135	142	160	173	144	195	132	165	138	145	139	140	179	157	SX7__H10.fsa	Shanxi
136	152	155	135	144	164	135	142	160	174	144	195	132	165	138	145	139	140	180	157	GJ17__E01.fsa	Gejiu_Yunnan
136	152	155	135	144	163	135	142	160	173	145	195	132	164	138	145	139	140	179	157	SX5__F10.fsa	Shanxi
136	152	155	135	144	164	135	142	160	168	141	195	132	164	138	145	143	140	179	157	QNSG12	Qinghai
136	152	155	135	144	164	135	142	160	168	142	195	132	164	138	145	143	140	182	157	GJ14__C01.fsa	Gejiu_Yunnan
136	152	155	135	144	164	135	142	160	168	142	195	132	165	138	145	143	140	182	0	GJ16__D01.fsa	Gejiu_Yunnan
136	152	155	135	144	164	135	143	160	174	143	194	132	165	137	145	143	139	185	157	YM32__F08.fsa	Yimen_Yunnan
136	152	155	135	144	164	135	142	160	174	143	194	132	165	137	145	143	139	185	157	YM35__G08.fsa	Yimen_Yunnan
136	152	152	135	144	164	135	142	160	156	143	194	132	165	137	145	143	139	179	157	HB15__B02.fsa	Hubei
136	152	155	135	144	164	135	142	160	168	143	194	132	165	138	131	143	140	185	157	HB10__F01.fsa	Hubei
136	152	155	135	144	164	135	142	160	0	141	195	0	164	138	145	0	140	160	157	HEN06	Henan
136	152	152	135	144	173	135	142	160	150	143	195	132	164	138	145	139	140	179	157	DB6__G03.fsa	Jilin
136	152	152	135	144	173	135	142	160	150	143	195	132	164	138	145	139	140	179	157	DB7__H03.fsa	Jilin
136	152	152	135	144	173	135	142	160	150	142	195	132	164	138	145	139	140	173	157	DB10__C04.fsa	Jilin
136	152	152	135	144	173	135	142	160	150	142	195	132	164	138	145	139	140	173	157	NH18__E05.fsa	Neimenggu
136	152	152	135	144	173	135	142	160	150	142	195	132	164	138	145	139	140	173	157	NH21__F05.fsa	Neimenggu
136	152	152	135	144	173	135	142	160	150	142	195	132	164	138	145	139	140	173	157	NH5__A05.fsa	Neimenggu
136	152	152	135	144	173	135	142	160	150	142	195	132	164	138	145	139	140	173	157	NH6__B05.fsa	Neimenggu
136	152	152	135	144	173	135	142	160	150	142	195	132	164	138	145	139	140	173	157	NH9__C05.fsa	Neimenggu
136	152	149	135	144	173	135	142	160	150	142	195	132	164	138	145	139	140	179	157	GD02	Guangdong
136	152	149	135	144	173	135	141	160	150	143	195	132	164	138	145	139	140	179	157	DB2__C03.fsa	Jilin
136	152	152	135	144	173	135	141	160	150	143	195	132	164	137	145	139	139	179	157	DB4__E03.fsa	Jilin
136	152	152	135	144	173	135	141	160	150	143	195	132	164	137	145	139	139	179	157	DB9__B04.fsa	Jilin
136	152	149	135	144	173	135	142	160	150	143	195	132	164	137	145	139	139	179	157	DB5__F03.fsa	Jilin
136	152	152	135	144	173	135	142	160	150	142	195	132	164	137	145	139	139	173	157	DB13__F04.fsa	Jilin
136	152	152	135	144	173	135	142	160	150	141	195	132	164	137	145	139	139	173	157	NM4__H06.fsa	Neimenggu
136	152	152	135	0	173	0	142	0	150	142	0	132	164	138	145	139	140	173	157	NH10__D05.fsa	Neimenggu
136	152	152	135	144	173	135	142	160	150	142	196	132	164	0	0	139	0	173	0	DB11__D04.fsa	Jilin
136	152	155	135	144	164	135	142	160	168	142	195	132	164	137	145	150	139	179	157	ZSS03	Zhejiang
136	152	155	135	144	164	135	142	160	168	142	194	132	165	137	145	150	139	179	157	HB20__D02.fsa	Hubei
136	152	152	135	144	164	135	142	160	168	142	195	132	164	137	145	139	139	187	157	DB1__B03.fsa	Jilin
136	152	152	135	144	164	135	142	160	168	142	195	132	165	137	145	139	139	187	157	DB3__D03.fsa	Jilin
136	152	155	135	144	164	135	143	160	168	142	195	132	164	137	145	143	139	176	157	HB21__E02.fsa	Hubei
136	152	155	135	144	164	135	143	160	168	142	195	132	165	137	145	143	139	176	157	HB25__A03.fsa	Hubei
136	152	156	135	144	164	135	142	160	168	141	195	132	164	137	145	143	139	179	157	ZSS01	Zhejiang
0	152	156	135	144	164	135	142	160	174	142	196	132	164	137	145	143	139	182	157	QNSG6	Qinghai
136	152	156	135	144	164	135	143	160	174	144	195	132	164	137	146	139	139	179	157	SX11__D11.fsa	Shanxi
136	152	156	136	144	164	135	142	160	168	143	195	132	164	138	146	150	139	179	157	HB24__H02.fsa	Hubei
136	152	152	135	144	163	135	0	160	156	0	195	132	154	138	145	139	140	187	157	DB12__E04.fsa	Jilin
136	152	155	135	144	0	135	0	160	156	0	195	132	0	138	145	139	140	176	153	DL4__B06.fsa	Dianchi_Yunnan
136	152	155	135	144	164	135	141	160	156	143	194	132	165	0	0	143	0	167	0	GX8__B05.fsa	Guangxi
136	152	152	135	144	164	135	142	160	156	143	194	132	165	0	0	143	0	179	0	HB13__H01.fsa	Hubei
136	152	155	135	144	163	135	142	160	156	142	194	132	165	0	0	143	0	178	0	GX14__D05.fsa	Guangxi
136	152	155	135	144	164	135	142	160	156	142	194	132	165	0	0	143	0	179	0	GX9__C05.fsa	Guangxi
136	152	155	135	144	164	135	142	160	173	142	194	132	165	0	0	143	0	185	0	YM39__B09.fsa	Yimen_Yunnan
136	152	155	135	144	161	135	142	160	156	142	195	132	164	0	0	143	0	176	0	XJzwy01	Xinjiangzwy
136	152	155	135	144	161	135	142	160	156	142	195	132	164	0	0	143	0	176	0	DL14__B07.fsa	Dianchi_Yunnan
136	152	155	135	144	161	135	141	160	178	143	194	0	164	0	0	0	0	0	0	GX45__C06.fsa	Guangxi
136	152	155	135	142	164	0	142	0	168	143	0	132	165	138	145	139	140	167	157	GX52__A07.fsa	Guangxi
136	152	155	135	0	164	0	142	0	168	142	0	131	165	138	145	150	140	179	157	HB23__G02.fsa	Hubei
136	152	155	135	0	163	0	142	160	168	143	199	132	165	138	145	143	140	179	157	SX18__C12.fsa	Shanxi
136	152	155	135	0	0	0	0	151	168	0	196	132	0	138	145	143	140	182	157	GJ10__A01.fsa	Gejiu_Yunnan
136	152	155	135	144	163	135	143	160	156	144	194	135	165	138	0	142	140	167	155	GX49__G06.fsa	Guangxi
0	0	0	0	144	164	135	142	160	174	144	195	132	165	138	145	139	140	179	157	GJ23__A02.fsa	Gejiu_Yunnan
0	0	0	0	0	164	0	142	0	173	145	0	132	165	138	145	139	140	179	157	GJ7__F12.fsa	Gejiu_Yunnan
136	152	156	135	151	161	145	140	154	157	143	188	133	164	132	146	139	139	153	157	GD04	Guangdong
0	152	156	136	142	0	136	141	160	157	143	193	132	161	135	129	143	139	176	157	XJzwy06	Xinjiangzwy
0	0	0	0	140	149	141	139	163	0	143	193	0	164	135	145	0	139	0	153	HN14__F03.fsa	Hainan
0	0	0	0	144	149	141	143	163	0	142	195	0	164	134	145	0	142	159	153	YM19__D08.fsa	Yimen_Yunnan
136	152	152	135	144	149	141	143	163	0	141	195	0	164	134	145	0	142	0	153	YNhj11	Heijing_Yunnan
136	152	152	135	144	149	141	143	163	0	141	195	0	164	134	129	0	142	159	153	YNhj12	Heijing_Yunnan
136	152	152	135	144	149	141	143	163	0	142	195	0	164	0	0	0	0	0	0	YM5__E07.fsa	Yimen_Yunnan
136	152	152	135	144	149	141	143	163	143	142	195	132	164	134	128	135	142	166	153	YM14__A08.fsa	Yimen_Yunnan
136	152	152	135	144	149	141	143	163	143	142	195	132	164	134	128	135	142	166	153	YM23__E08.fsa	Yimen_Yunnan
136	152	152	135	144	149	141	143	163	143	142	195	132	164	134	145	135	142	167	153	YM4__D07.fsa	Yimen_Yunnan
136	159	152	135	140	149	141	141	163	150	144	193	125	164	135	145	139	139	183	153	HN24__H04.fsa	Hainan
136	159	152	135	140	149	141	141	163	150	144	193	125	164	135	145	139	139	183	153	HN7__H02.fsa	Hainan
0	159	152	135	140	149	141	141	163	150	144	193	125	164	135	145	139	139	183	153	HN17__A04.fsa	Hainan
136	159	152	135	140	149	141	139	163	150	143	193	125	164	135	145	139	139	183	153	HN18__B04.fsa	Hainan
136	159	152	135	140	149	141	139	163	150	143	193	125	164	135	145	139	139	183	153	HN4__E02.fsa	Hainan
136	159	152	135	140	149	141	139	163	150	143	193	125	164	135	145	139	139	183	153	HN6__G02.fsa	Hainan
136	159	152	135	140	149	141	140	163	150	143	193	123	164	135	145	137	139	183	153	HN15__G03.fsa	Hainan
136	159	152	135	140	149	141	140	163	150	143	193	123	164	135	145	137	139	183	153	HN21__E04.fsa	Hainan
136	159	152	135	140	149	141	139	163	150	143	193	123	164	135	145	137	139	183	153	HN9__B03.fsa	Hainan
136	159	152	138	140	149	141	139	163	150	143	193	133	164	135	145	137	139	166	153	HN1__C02.fsa	Hainan
136	159	152	139	140	149	141	141	163	150	144	193	135	164	135	145	139	139	166	153	HN2__D02.fsa	Hainan
0	159	146	0	140	0	141	0	163	150	0	193	123	0	135	145	137	139	183	153	HN10__C03.fsa	Hainan
136	159	153	136	140	149	145	141	163	150	142	193	132	164	134	145	137	138	183	153	HN16__H03.fsa	Hainan
136	159	152	135	139	149	141	139	160	150	141	194	132	164	135	145	137	139	168	153	ZSS05	Zhejiang
136	159	152	135	146	149	141	140	160	137	142	194	132	164	135	145	137	139	168	153	GZ05	Guizhou
136	159	152	135	144	149	141	140	160	137	142	194	132	164	134	145	137	138	180	153	YNhj04	Heijing_Yunnan
136	159	152	135	144	149	141	140	160	150	142	194	132	164	134	145	137	138	180	153	YNhj16	Heijing_Yunnan
136	159	152	135	144	149	141	140	160	150	142	194	132	164	134	145	138	138	180	153	YNhj07	Heijing_Yunnan
136	159	152	135	144	149	141	139	160	182	141	194	0	164	135	145	0	139	0	153	GZ03	Guizhou
136	159	152	135	144	149	141	139	160	0	141	194	0	164	135	145	0	139	160	153	GZ04	Guizhou
136	159	152	135	144	149	141	140	160	0	142	194	0	164	135	145	0	139	0	153	GZ01	Guizhou
136	159	152	135	144	149	141	140	160	182	142	194	0	164	135	145	0	139	0	153	GZ02	Guizhou
136	159	152	135	144	149	141	140	160	0	142	194	0	164	135	145	0	139	160	153	GZ07	Guizhou
136	159	152	135	144	149	141	140	160	182	142	194	0	164	134	145	0	138	0	153	GZ06	Guizhou
136	152	152	135	144	161	135	140	160	137	142	194	132	164	138	145	143	139	168	153	GD05	Guangdong
136	159	152	135	0	149	0	139	151	150	143	192	125	164	0	0	139	0	183	0	HN20__D04.fsa	Hainan
136	152	153	136	144	149	142	144	163	153	142	197	139	164	137	145	148	142	162	153	YM18__C08.fsa	Yimen_Yunnan
136	152	155	120	151	126	145	141	154	138	165	188	139	164	134	126	131	140	153	150	HN12__E03.fsa	Hainan
136	152	155	120	151	126	145	141	154	138	165	188	139	0	134	126	131	140	153	150	HN8__A03.fsa	Hainan
0	0	0	0	151	126	145	141	154	0	165	188	0	164	134	126	0	140	149	150	HN5__F02.fsa	Hainan
0	0	0	0	151	148	145	141	154	138	165	186	139	0	134	126	131	131	157	150	JZG09	Sichuan
0	0	0	0	151	148	145	141	154	138	165	186	139	0	134	126	131	131	157	150	JZG11	Sichuan
0	0	149	0	151	148	145	141	154	137	165	186	138	164	134	126	130	131	157	150	JZG07	Sichuan
136	145	155	120	151	148	145	141	154	137	165	186	138	164	134	126	130	131	157	150	JZG03	Sichuan
136	145	155	120	151	148	145	141	154	137	165	186	138	164	134	126	130	131	157	150	JZG04	Sichuan
136	145	155	120	151	148	145	141	154	137	165	186	138	164	134	126	130	131	157	150	JZG05	Sichuan
136	145	155	120	151	148	145	141	154	137	165	186	138	0	134	126	130	131	157	150	DL8__F06.fsa	Dianchi_Yunnan
136	145	155	120	151	149	145	141	154	137	165	186	138	0	134	126	130	131	157	150	JZG06	Sichuan
136	145	155	120	151	148	145	141	154	138	165	186	139	0	134	126	131	131	157	150	JZG10	Sichuan
136	145	155	120	151	148	145	140	154	137	162	186	132	0	134	126	130	131	157	150	JZG12	Sichuan
136	145	155	120	151	148	145	141	154	138	165	186	139	164	0	0	131	0	157	0	JZG01	Sichuan
136	153	155	120	152	126	145	141	154	0	164	188	0	165	134	126	0	140	150	150	YNhj03	Heijing_Yunnan
136	153	155	120	152	126	145	141	154	0	164	188	0	165	134	126	0	140	153	151	YNhj14	Heijing_Yunnan
136	153	155	120	151	126	145	141	154	0	165	188	0	164	134	126	0	140	0	150	HN11__D03.fsa	Hainan
136	145	155	120	152	149	145	141	154	0	164	186	0	164	134	126	0	131	157	150	YNhj13	Heijing_Yunnan
136	145	155	120	152	149	145	141	154	0	164	186	0	165	134	126	0	131	0	150	YNhj10	Heijing_Yunnan
136	145	155	120	152	149	145	141	154	0	164	186	0	165	134	126	0	130	0	151	XZ04	Sichuan
136	145	155	120	152	149	145	141	154	136	164	186	0	170	134	126	130	131	157	150	YNhj02	Heijing_Yunnan
0	0	155	0	0	0	0	0	0	0	0	198	0	0	0	0	0	0	0	0	GZ08	Guizhou
141	156	152	139	144	161	136	0	162	143	142	197	142	164	0	0	142	0	155	0	DL11__G06.fsa	Dianchi_Yunnan
140	152	152	136	144	158	148	138	155	144	147	196	142	168	155	158	139	146	154	163	DL3__A06.fsa	Dianchi_Yunnan
140	152	152	136	144	158	148	138	155	144	147	198	140	168	155	158	139	146	154	163	DL6__D06.fsa	Dianchi_Yunnan
141	152	152	136	144	158	148	136	155	144	146	196	140	168	155	158	139	146	154	163	DL5__C06.fsa	Dianchi_Yunnan
140	152	155	135	144	158	148	144	155	144	146	195	142	168	152	158	139	146	157	163	HEN11	Henan
140	152	155	136	144	158	148	144	155	144	147	196	142	168	152	158	139	146	157	163	QNSG10	Qinghai
140	152	146	136	149	141	148	149	155	143	159	196	140	168	155	157	140	146	160	163	YM36__H08.fsa	Yimen_Yunnan
140	152	146	136	149	141	148	149	155	143	159	196	140	168	155	157	140	146	160	163	YM40__C09.fsa	Yimen_Yunnan
140	152	146	136	149	141	148	149	155	143	159	196	140	168	155	157	140	146	160	163	YM41__D09.fsa	Yimen_Yunnan
140	152	146	136	149	141	148	149	155	143	159	196	140	168	155	158	140	146	160	163	YM12__G07.fsa	Yimen_Yunnan
140	152	146	136	149	141	148	149	155	143	159	196	140	168	155	158	140	146	160	163	YM38__A09.fsa	Yimen_Yunnan
140	152	146	136	149	141	148	149	155	143	159	196	140	168	155	158	140	146	160	163	YM45__H09.fsa	Yimen_Yunnan
140	152	146	136	149	141	148	149	155	143	159	196	140	168	155	158	140	146	160	163	YM46__A10.fsa	Yimen_Yunnan
140	152	146	136	149	141	148	149	155	143	159	196	140	168	155	158	140	146	160	163	YM47__B10.fsa	Yimen_Yunnan
140	152	146	136	149	141	148	149	155	144	159	196	140	168	155	158	140	146	160	163	GJ11__B01.fsa	Gejiu_Yunnan
140	152	146	136	148	141	148	149	155	143	159	196	140	168	155	158	140	146	160	163	SX10__C11.fsa	Shanxi
141	152	146	136	149	141	148	149	155	143	159	196	140	168	155	158	140	146	160	163	GJ1__D12.fsa	Gejiu_Yunnan
141	152	146	136	149	141	148	149	155	143	159	196	140	168	155	158	140	146	160	163	GJ3__E12.fsa	Gejiu_Yunnan
141	152	146	136	148	141	148	149	155	143	159	196	140	168	155	158	140	146	160	163	GJ8__G12.fsa	Gejiu_Yunnan
140	152	146	135	149	141	148	149	155	143	159	196	140	168	155	158	140	146	160	163	YM2__C07.fsa	Yimen_Yunnan
140	152	146	135	149	141	148	149	155	143	159	196	140	168	155	158	140	146	160	163	YM43__F09.fsa	Yimen_Yunnan
140	152	146	135	149	141	148	149	155	143	158	196	140	168	155	158	140	146	160	163	XZ03	Sichuan
140	152	146	136	149	141	149	149	155	143	159	196	140	168	155	157	140	146	160	163	YM42__E09.fsa	Yimen_Yunnan
140	152	146	135	149	141	148	153	155	144	159	196	140	168	155	158	140	146	160	163	YM7__F07.fsa	Yimen_Yunnan
140	152	146	135	149	141	148	149	155	143	158	196	139	168	155	158	141	146	160	163	GZ09	Guizhou
140	152	146	136	149	141	148	149	155	143	159	196	140	168	0	0	140	0	160	0	YM44__G09.fsa	Yimen_Yunnan
140	152	146	135	149	141	148	149	149	143	158	196	141	168	155	154	140	141	160	163	XZ02	Sichuan
140	152	146	135	144	141	135	142	160	144	142	196	140	164	0	158	139	139	160	163	YM15__B08.fsa	Yimen_Yunnan
141	156	152	139	140	152	145	144	155	143	155	196	142	168	152	158	142	146	155	163	GJ24__B02.fsa	Gejiu_Yunnan
141	156	152	139	140	152	145	144	155	143	159	196	142	168	152	158	142	146	155	163	YM13__H07.fsa	Yimen_Yunnan
140	156	152	138	140	152	145	144	155	143	158	196	142	168	152	158	142	146	155	163	YNhj01	Heijing_Yunnan
140	156	152	138	140	152	145	144	155	144	158	196	142	168	152	154	139	146	155	163	YNhj05	Heijing_Yunnan
140	156	152	138	140	152	145	144	155	143	159	196	132	168	152	154	138	146	155	153	QNSG9	Qinghai
141	156	152	139	140	152	145	144	155	143	155	196	142	168	150	155	142	145	155	160	GJ9__H12.fsa	Gejiu_Yunnan
