# Supplementary material for: Historical Differentiation and Recent Hybridization in Natural Populations of the Nematode-Trapping Fungus Arthrobotrys oligospora in China
Source: Microorganisms. 2021 Sep 9;9(9):1919. doi: 10.3390/microorganisms9091919 (PMC8465350; doi:10.3390/microorganisms9091919)
Supplement: Supplementary file 1 [file microorganisms-09-01919-s001.zip › Figure S3 14 pairwise tanglegrams.pdf]

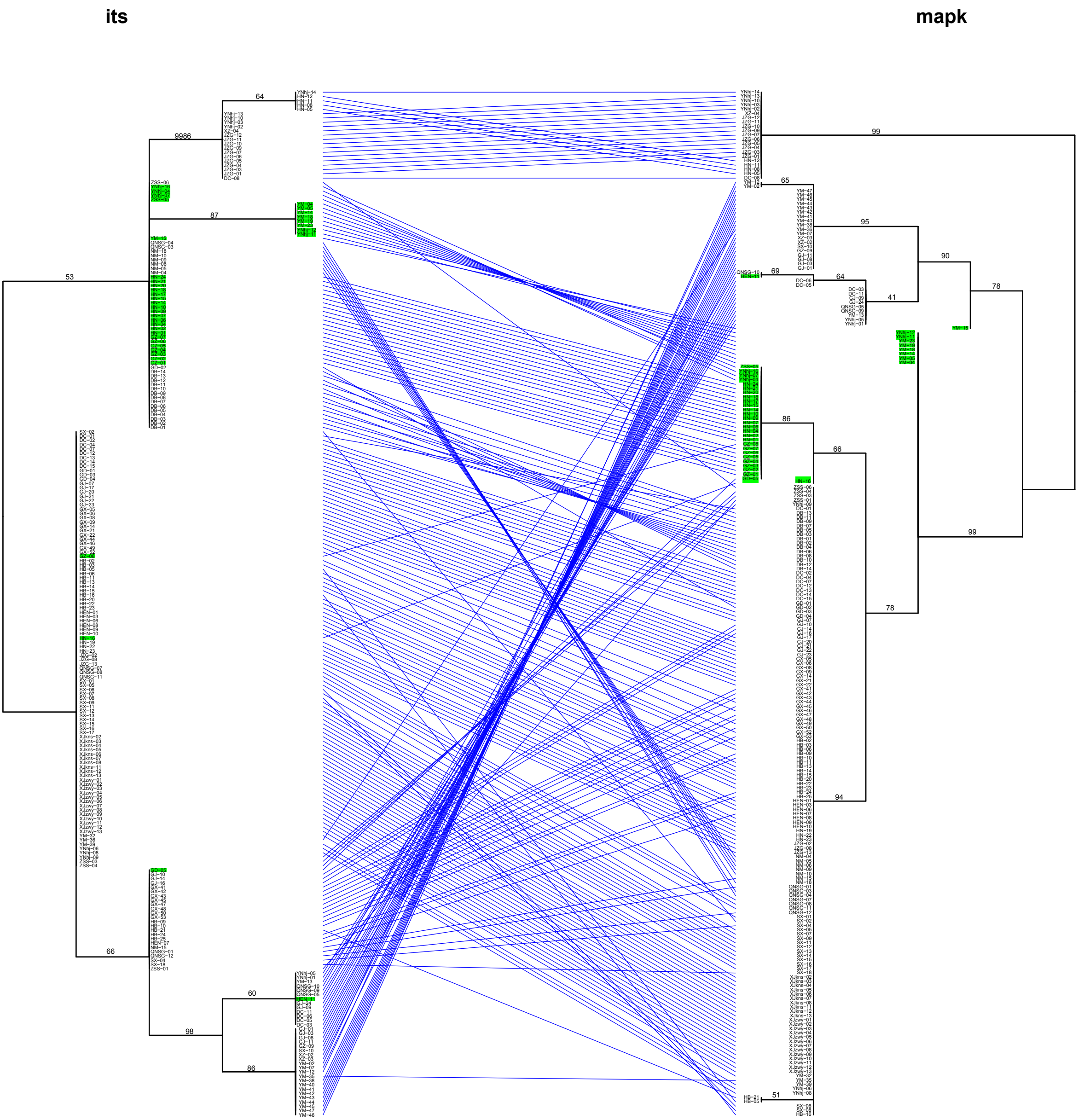
its
mapk

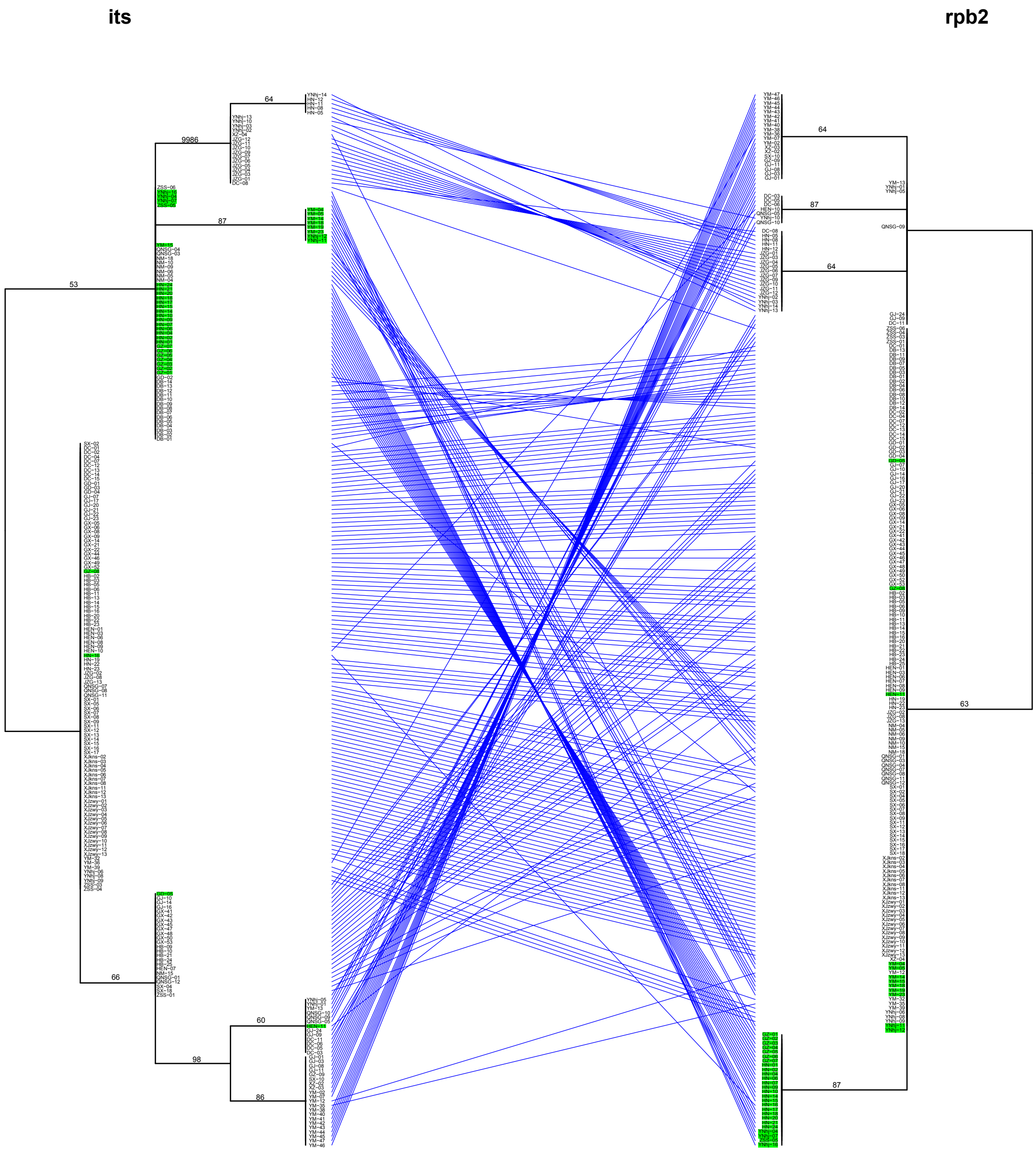
its
rpb2

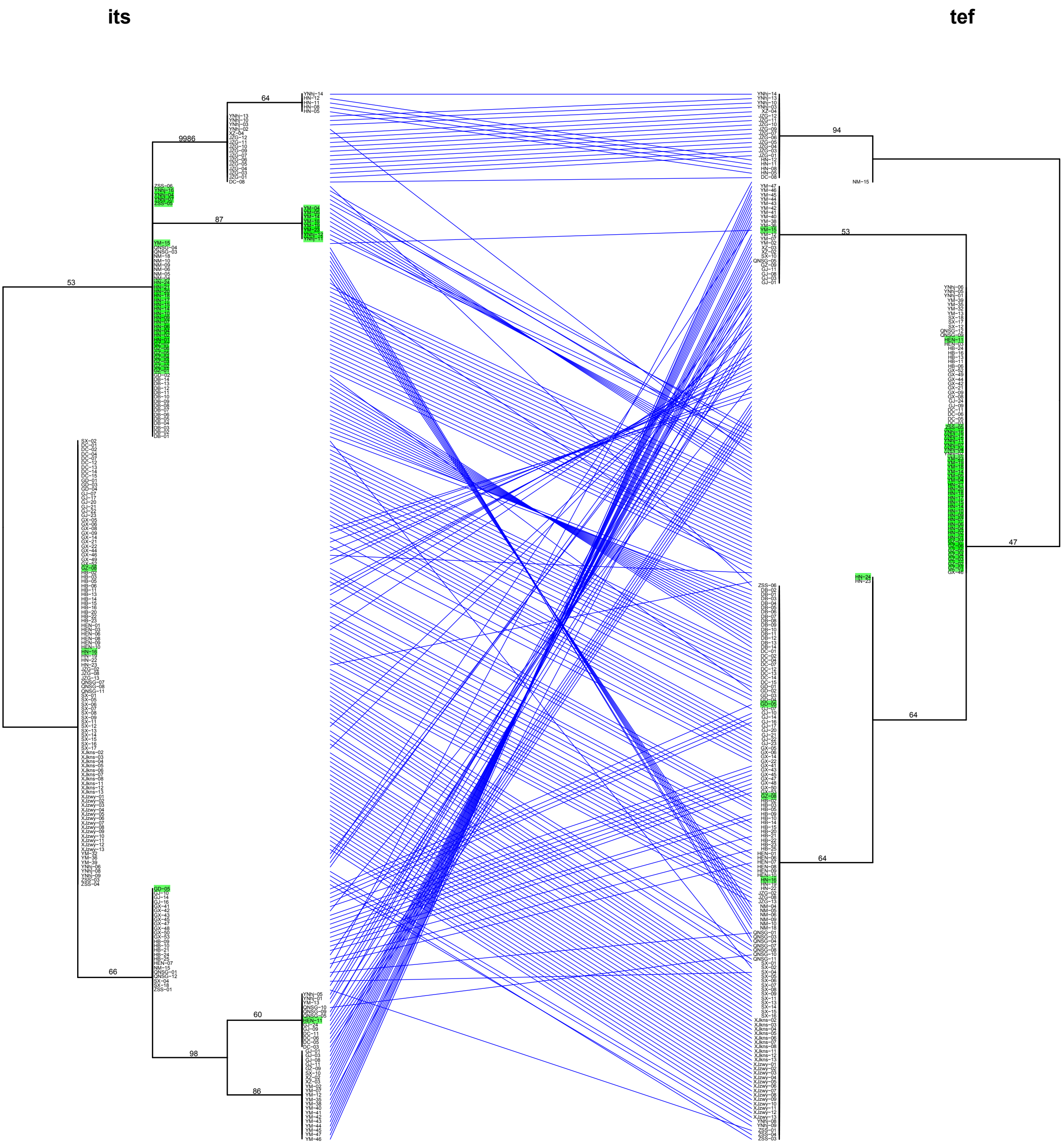
its
tef

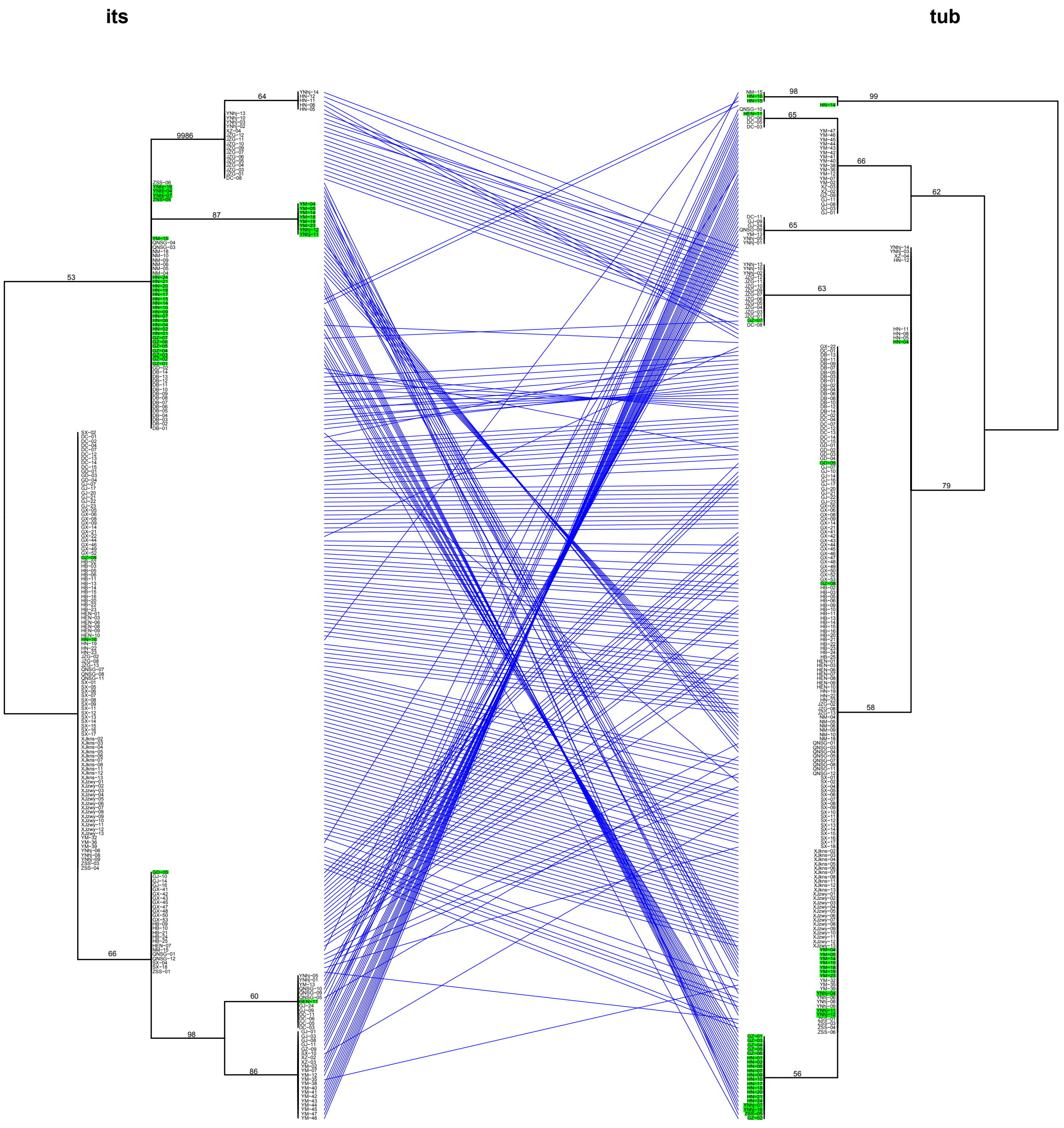
its
tub

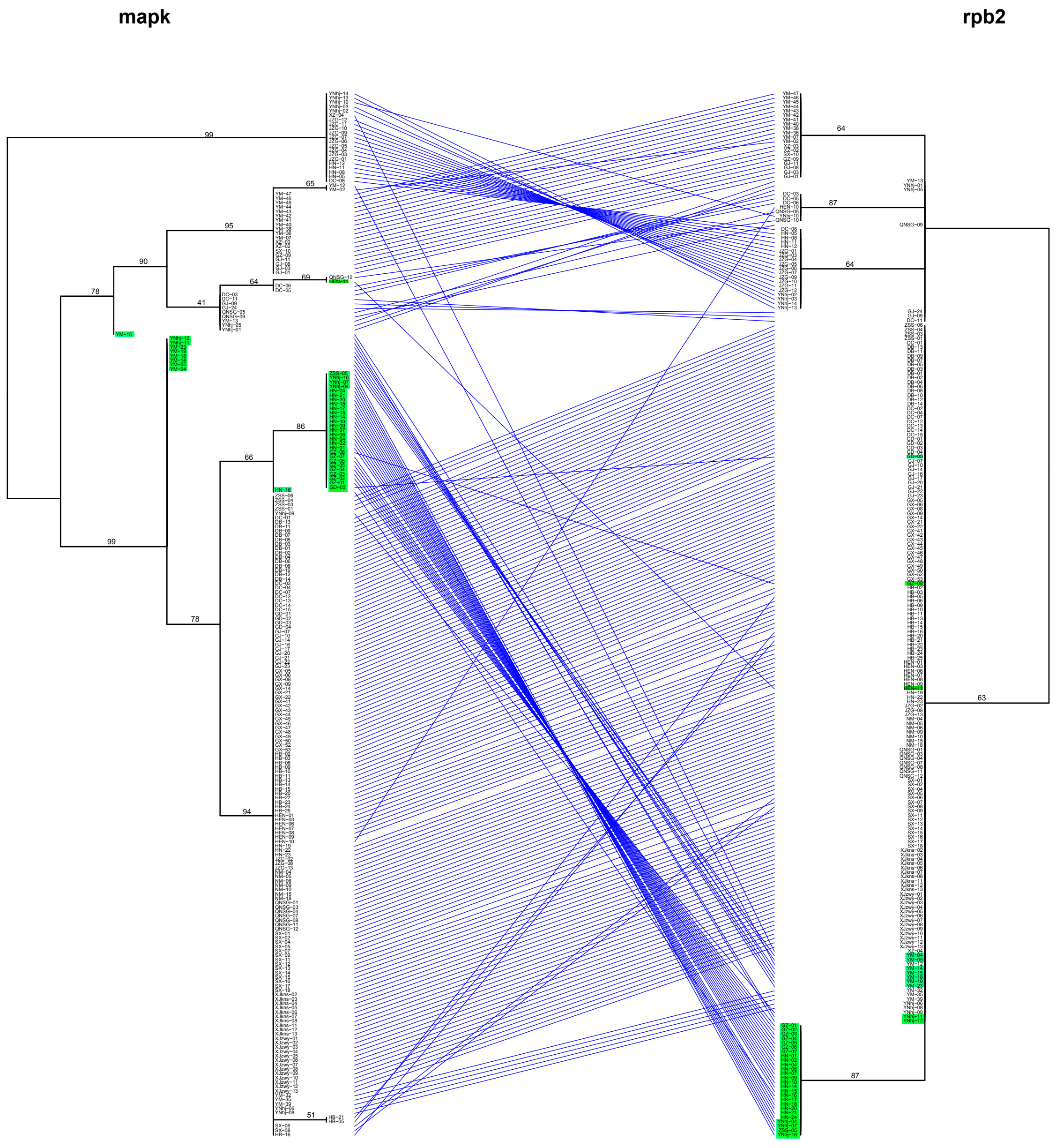
mapk
rpb2
99
65
95
90
78
41
64
69
86
66
99
78
94
51
64
87
64
63
87

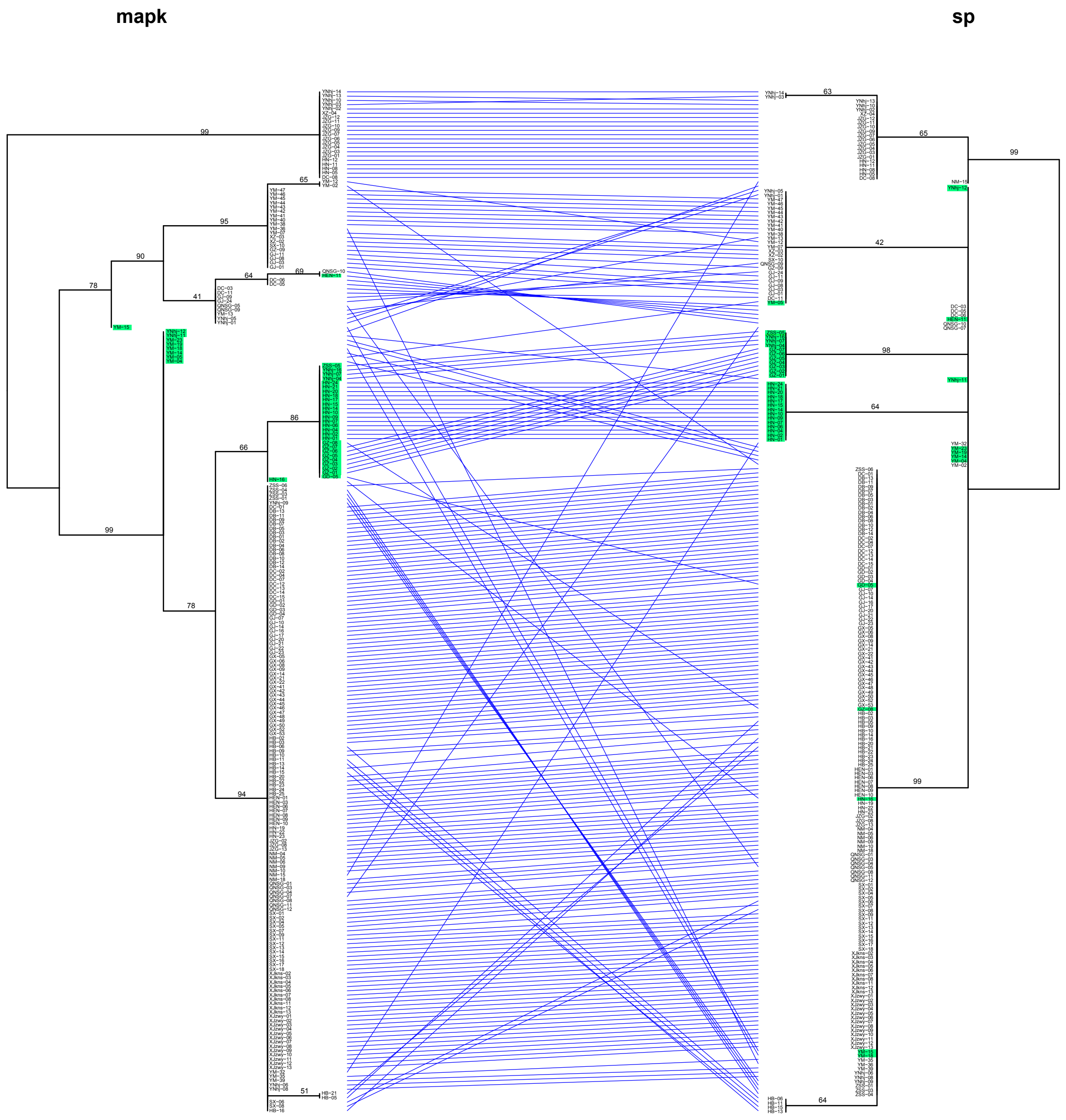
mapk
sp

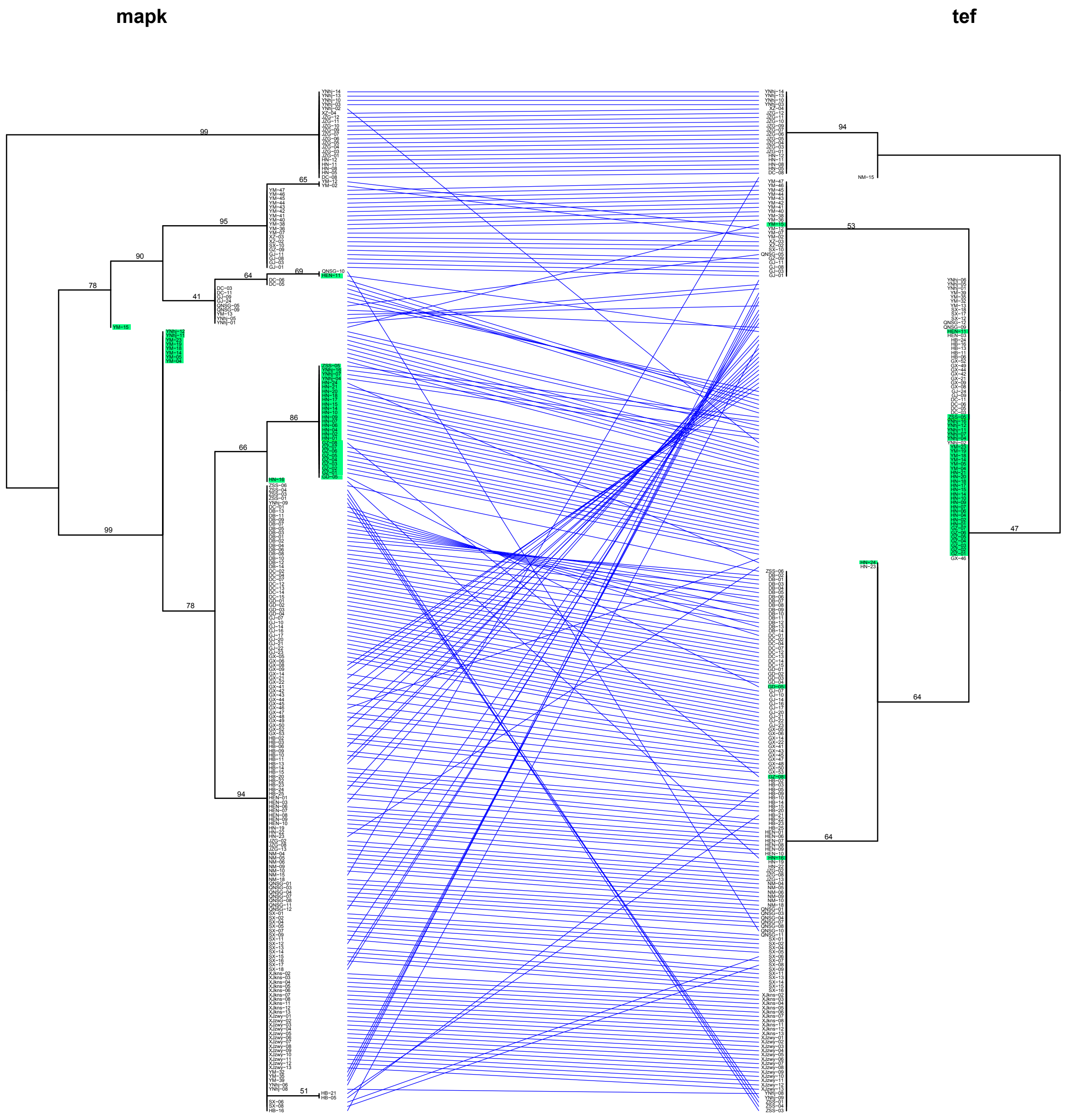
mapk
tef

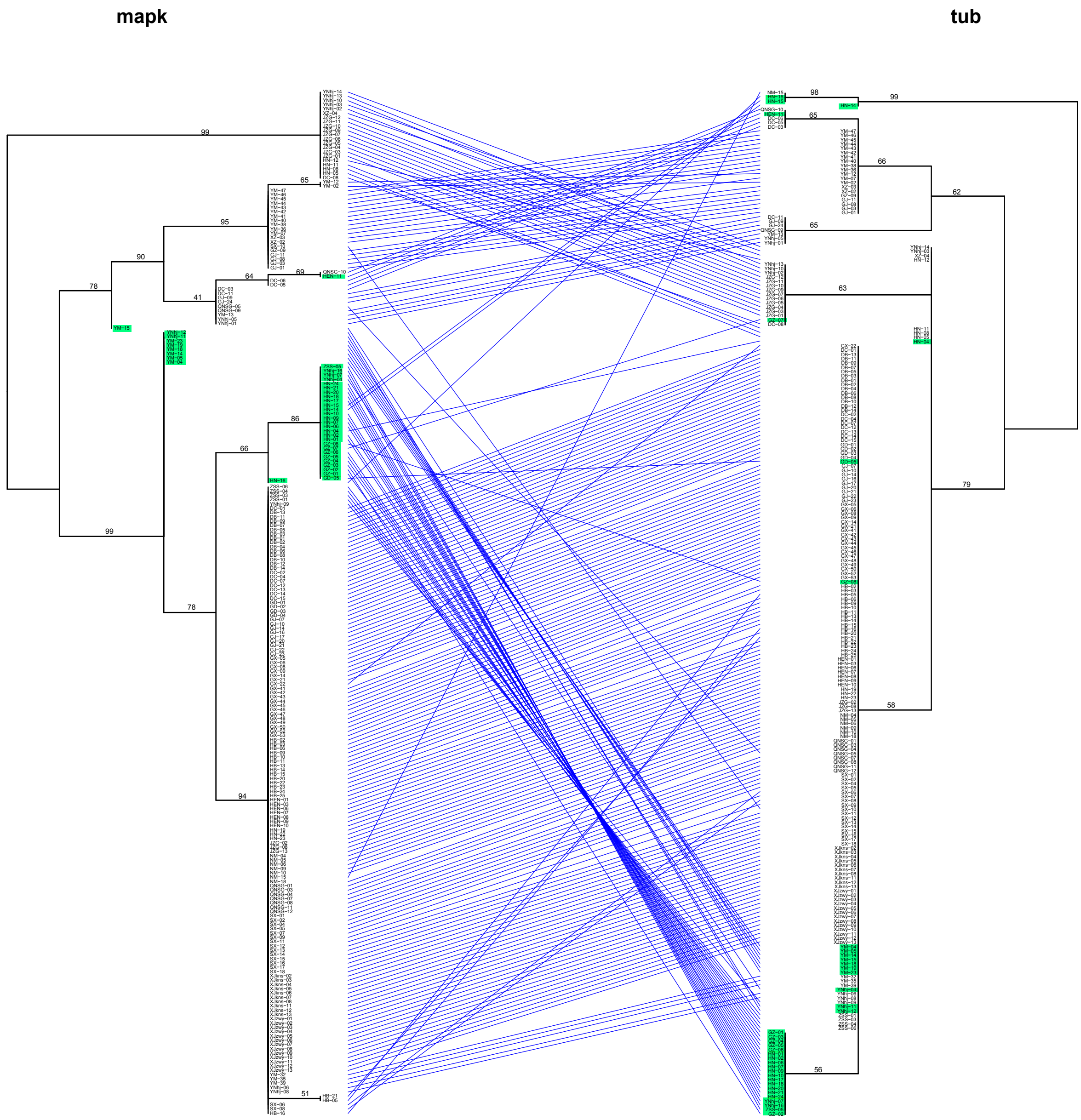
mapk
tub

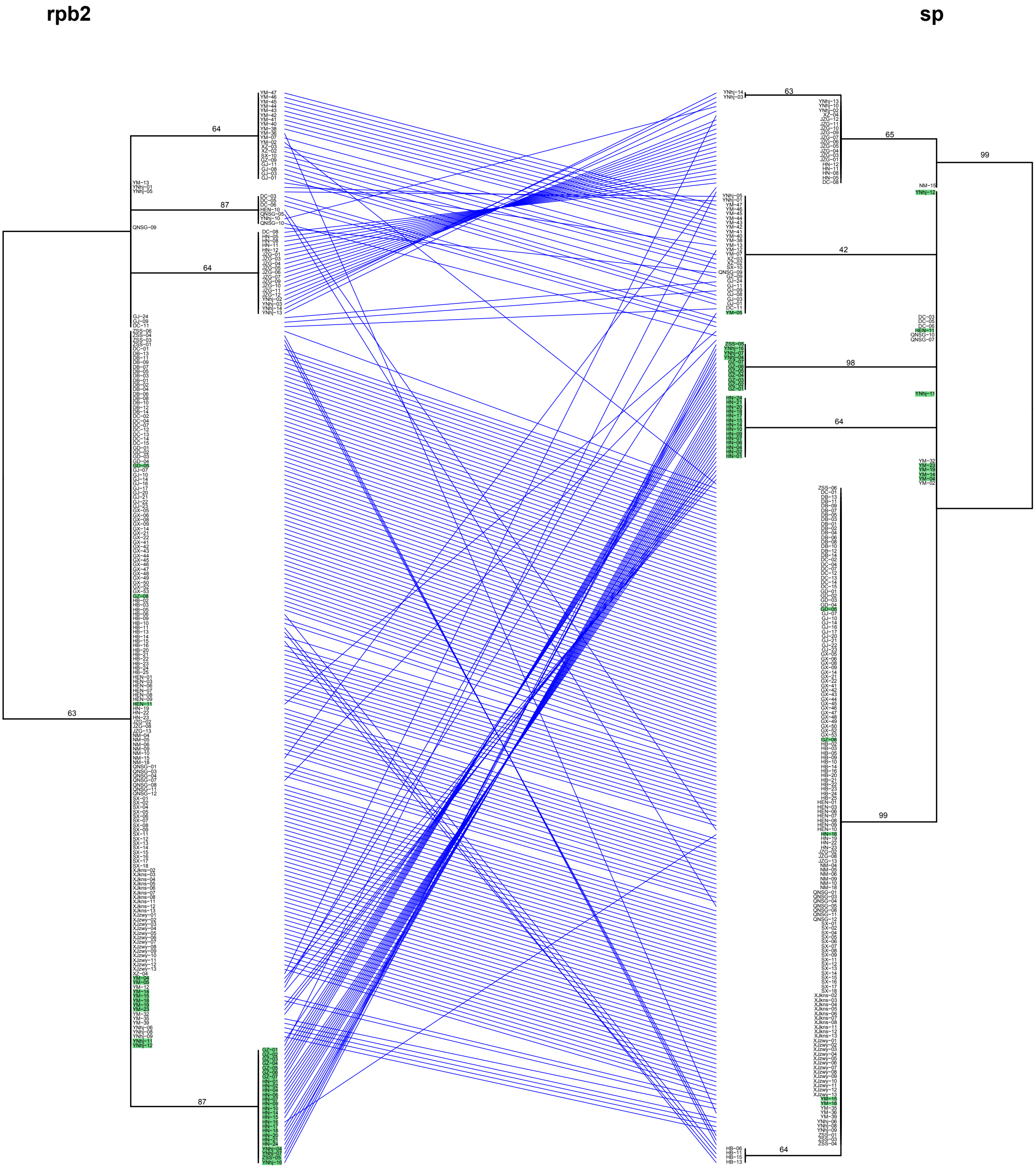
rpb2
sp

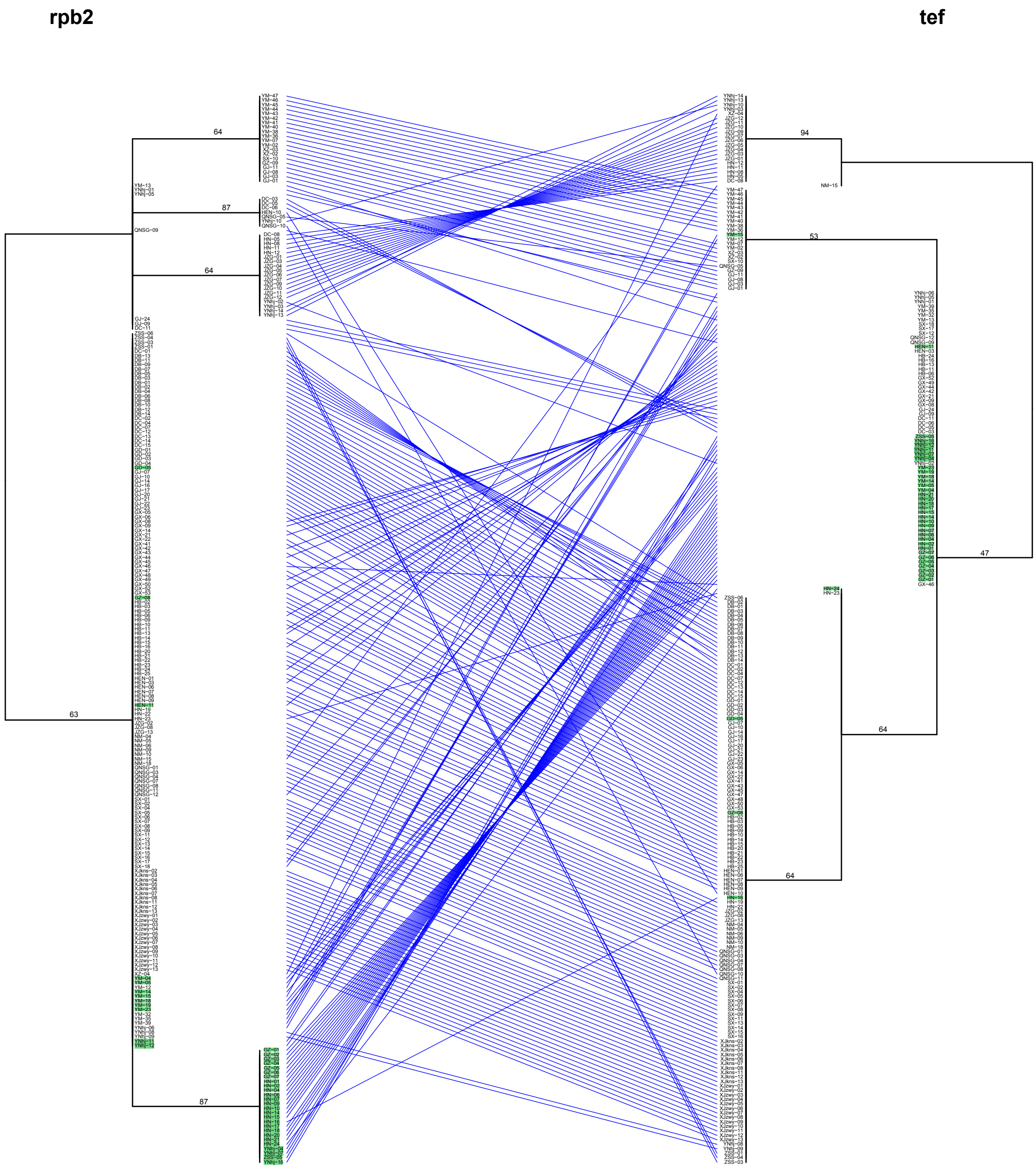
rpb2
tef
64
87
64
63
87
94
53
47
64
64

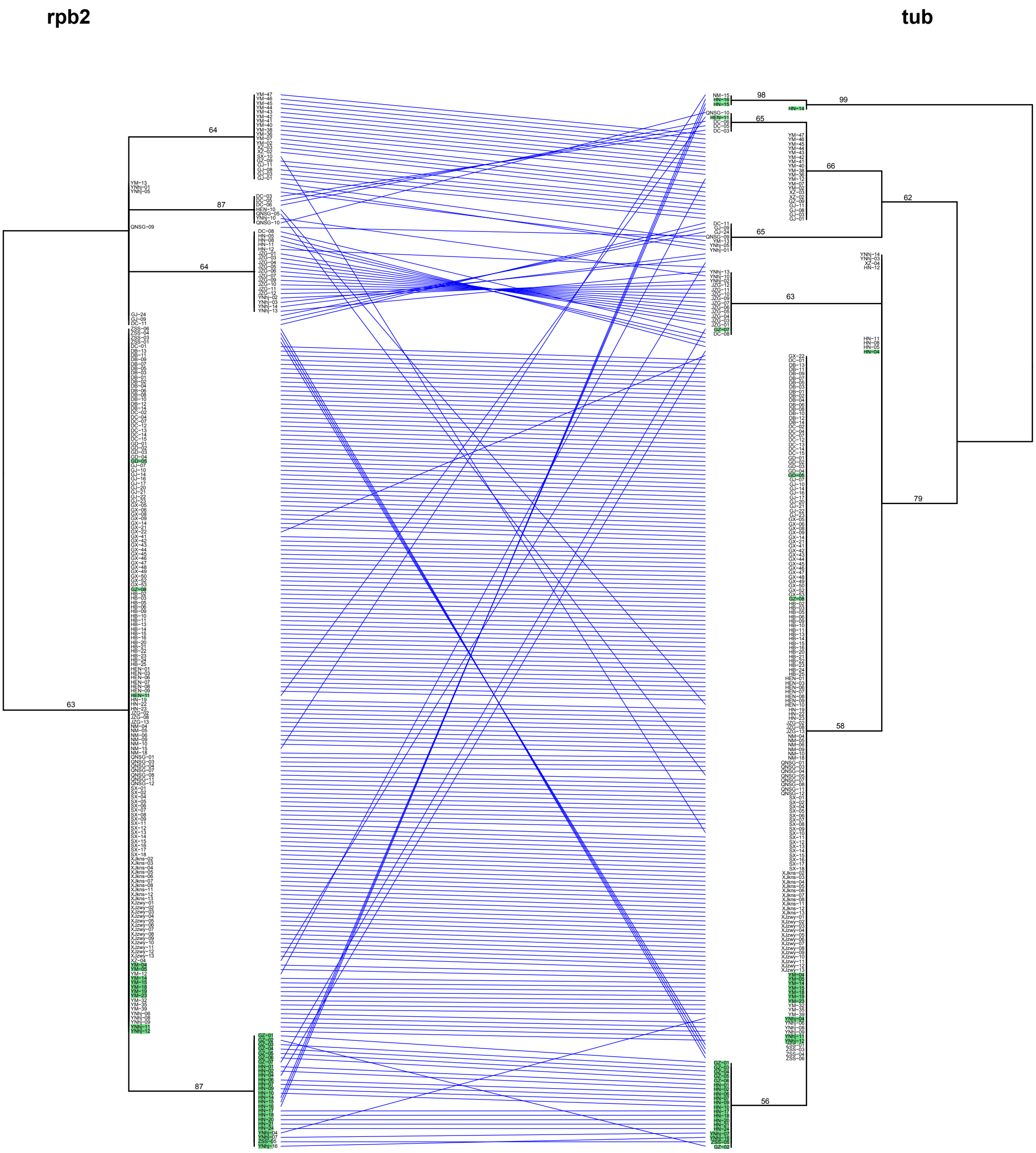

rpb2
tub
64
87
64
63
87
98
99
65
66
62
65
63
79
58
56

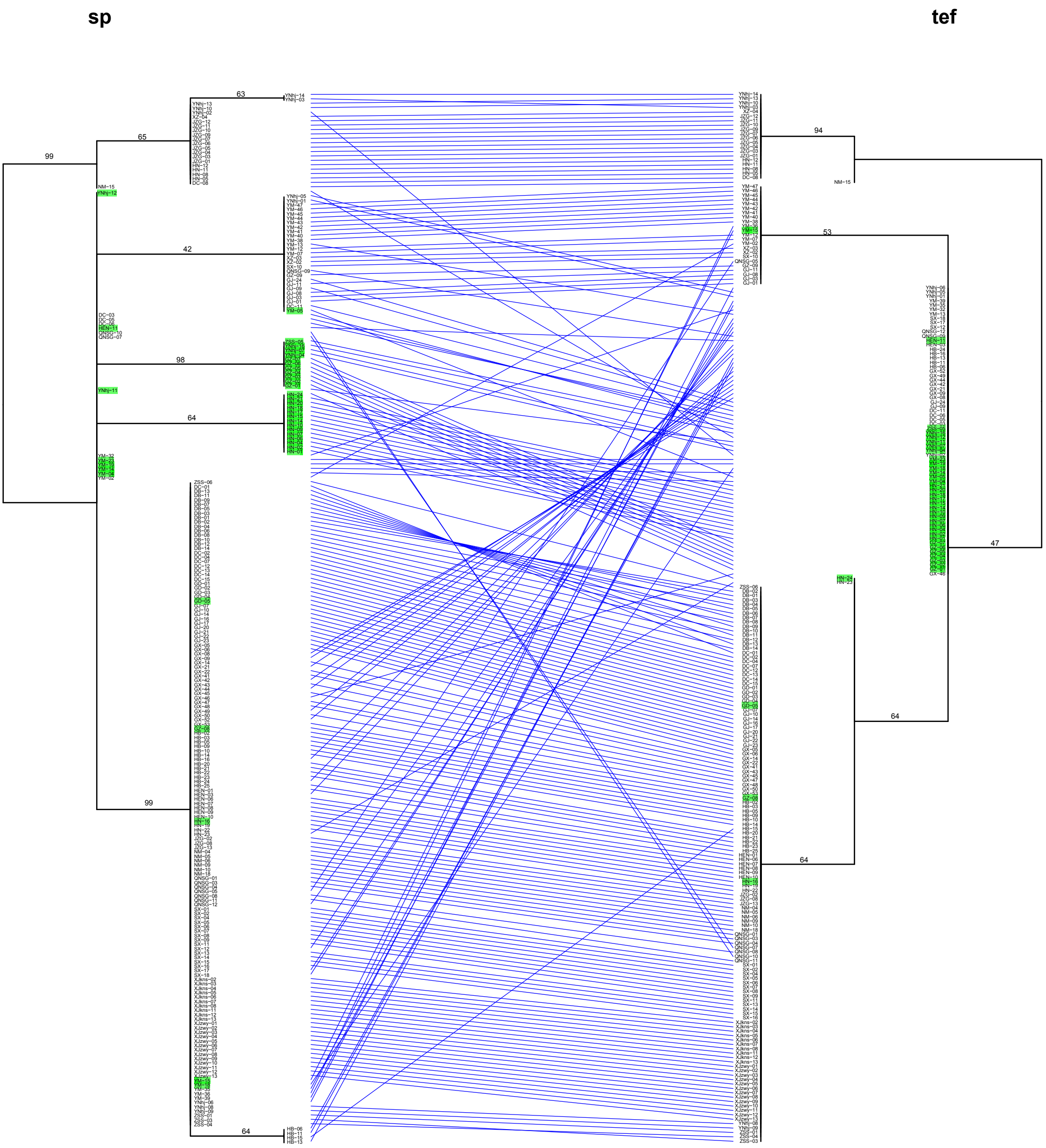

sp
tef
63
65
99
42
98
64
99
64
94
53
47
64
64

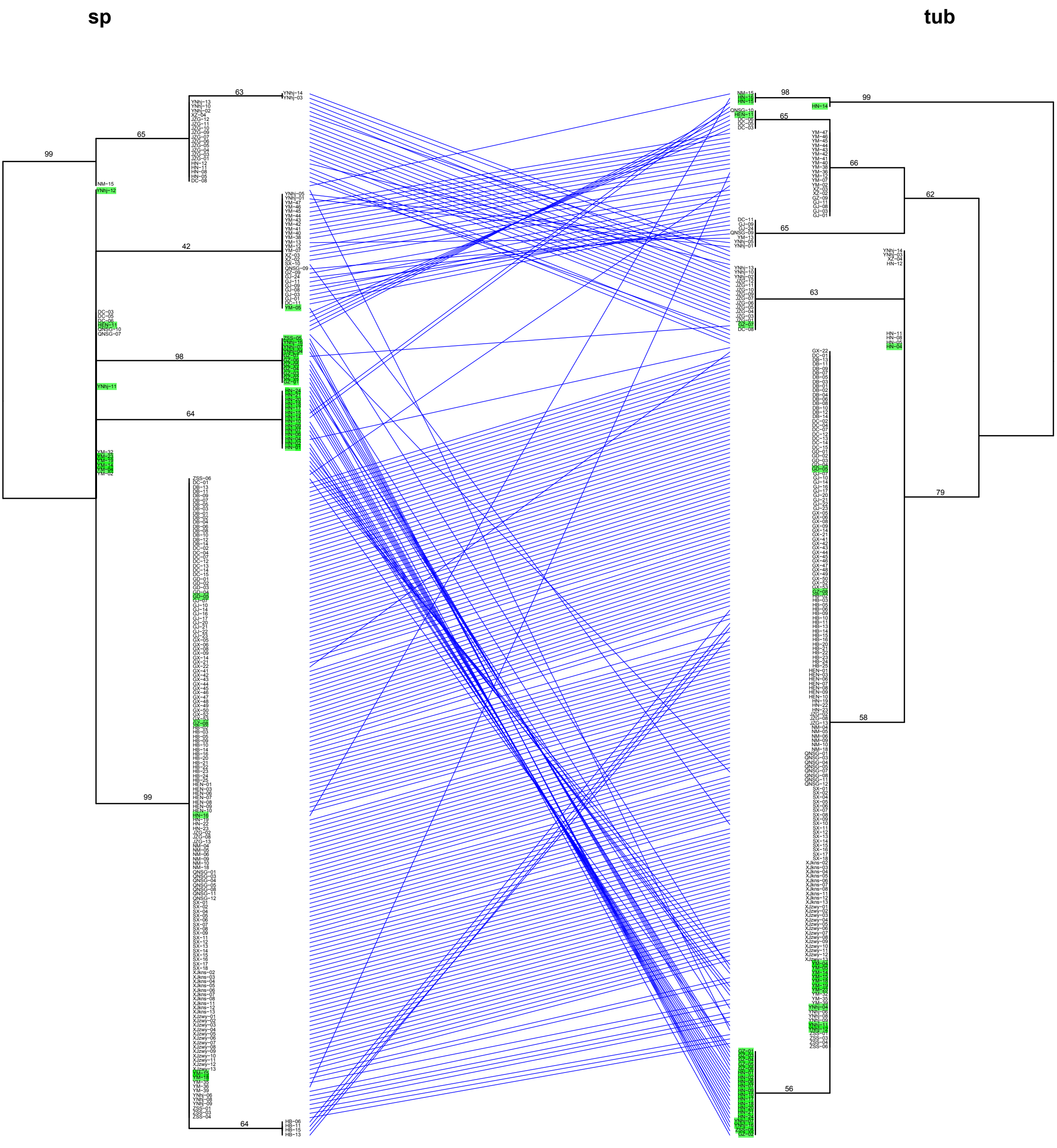
sp
tub

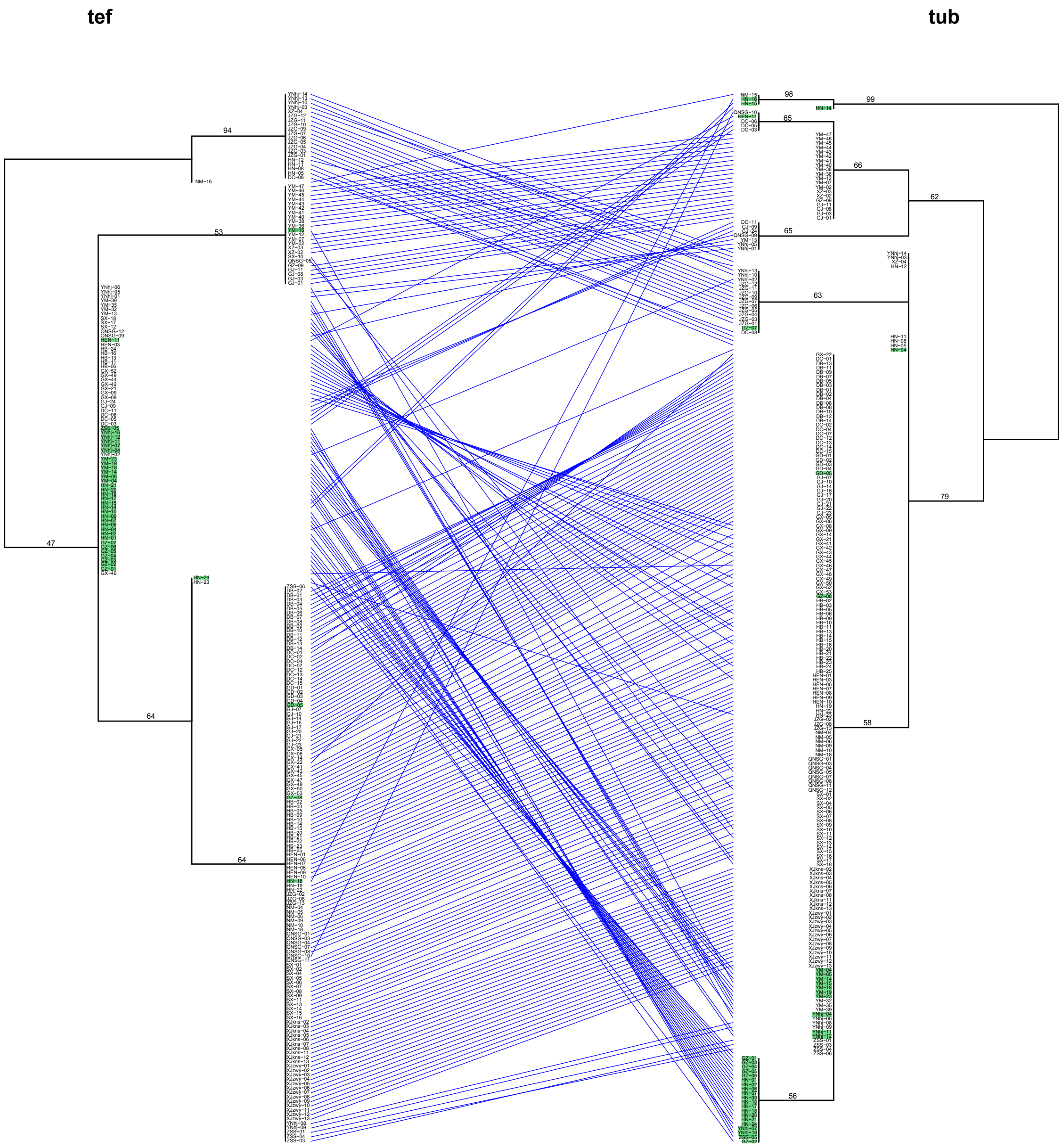
tef
tub
94
53
47
64
64
98
99
65
66
62
65
63
79
58
56
