# Supplementary material for: Historical Differentiation and Recent Hybridization in Natural Populations of the Nematode-Trapping Fungus Arthrobotrys oligospora in China
Source: Microorganisms. 2021 Sep 9;9(9):1919. doi: 10.3390/microorganisms9091919 (PMC8465350; doi:10.3390/microorganisms9091919)
Supplement: Supplementary file 1 [file microorganisms-09-01919-s001.zip › Table S1 Length and polymorphism information of six sequence fragments..pdf]

Table S1 Length and polymorphism information of six sequence fragments.

Sequence name	Length (bp)	No.of SNPs	Frequency
its	465	25	0.0538
sp	425	20	0.0471
mapk	422	42	0.0968
rpb2	184	7	0.0380
tub	322	24	0.0745
tef-1	288	7	0.0243
Total	2106	125	0.0594
