# Supplementary material for: Historical Differentiation and Recent Hybridization in Natural Populations of the Nematode-Trapping Fungus Arthrobotrys oligospora in China
Source: Microorganisms. 2021 Sep 9;9(9):1919. doi: 10.3390/microorganisms9091919 (PMC8465350; doi:10.3390/microorganisms9091919)
Supplement: Supplementary file 1 [file microorganisms-09-01919-s001.zip › Table S2 Pairwise differentiations A. oligospora isolates from 16 geographic populations in China based on MLST dataset.pdf]

Table S2 Pairwise differentiations *A. oligospora* isolates from 16 geographic populations in China based on MLST dataset.

HuB	HeN	ZheJ	NeiM	ShanX	QingH	GuangD	GuangX	HaiN	Dianchi_YunN	GeJ_YunN	YiM_YunN	HeiJ_YunN	GuiZ	SiC	Tibet	
	0.282	0.073	0.093	0.419	0.004	0.139	0.388	0.001	0.006	0.061	0.001	0.005	0.003	0.185	0.004	HuB
0.063		0.393	0.192	0.472	0.257	0.367	0.421	0.063	0.181	0.154	0.186	0.062	0.076	0.298	0.022	HeN
0.156	0.000		0.280	0.279	0.367	0.528	0.134	0.254	0.049	0.364	0.046	0.099	0.153	0.334	0.030	ZheJ
0.264	0.000	0.000		0.301	0.323	0.605	0.144	0.441	0.115	0.337	0.050	0.092	0.072	0.244	0.104	NeiM
0.004	0.000	0.000	0.049		0.331	0.289	0.477	0.006	0.044	0.372	0.045	0.028	0.009	0.224	0.014	ShanX
0.231	0.000	0.053	0.067	0.015		0.408	0.090	0.005	0.227	0.274	0.198	0.028	0.081	0.270	0.039	QingH
0.123	0.000	0.000	0.000	0.000	0.025		0.149	0.355	0.151	0.383	0.055	0.054	0.114	0.386	0.086	GuangD
0.000	0.000	0.094	0.149	0.000	0.142	0.149		0.008	0.057	0.161	0.007	0.018	0.001	0.276	0.016	GuangX
0.346	0.144	0.059	0.000	0.223	0.182	0.076	0.259		0.025	0.041	0.002	0.050	0.219	0.175	0.035	HaiN
0.546	0.169	0.310	0.221	0.329	0.066	0.299	0.438	0.243		0.278	0.186	0.183	0.054	0.238	0.358	Dianchi_YunN
0.415	0.000	0.150	0.078	0.082	0.000	0.135	0.300	0.171	0.000		0.378	0.144	0.216	0.551	0.406	GeJ_YunN
0.376	0.097	0.212	0.204	0.171	0.034	0.213	0.297	0.229	0.060	0.000		0.009	0.103	0.183	0.166	YiM_YunN
0.359	0.180	0.158	0.139	0.247	0.179	0.169	0.281	0.117	0.071	0.131	0.187		0.159	0.532	0.196	HeiJ_YunN
0.545	0.203	0.130	0.220	0.305	0.172	0.223	0.453	0.045	0.191	0.103	0.141	0.113		0.105	0.127	GuiZ
0.569	0.130	0.175	0.000	0.280	0.148	0.148	0.431	0.076	0.000	0.028	0.209	0.000	0.182		0.410	SiC
0.736	0.389	0.493	0.356	0.505	0.299	0.492	0.647	0.353	0.000	0.031	0.144	0.113	0.293	0.000		Tibet
