# Supplementary material for: Historical Differentiation and Recent Hybridization in Natural Populations of the Nematode-Trapping Fungus Arthrobotrys oligospora in China
Source: Microorganisms. 2021 Sep 9;9(9):1919. doi: 10.3390/microorganisms9091919 (PMC8465350; doi:10.3390/microorganisms9091919)
Supplement: Supplementary file 1 [file microorganisms-09-01919-s001.zip › Table S3 Pairwise differentiations A. oligospora isolates from 19 geographic populations in China based on STR dataset..pdf]

Table S3 Pairwise differentiations *A. oligospora* isolates from 19 geographic populations in China based on STR dataset.

HuB	HeN	ZheJ	NeiM	ShanX	JiL	QingH	XinJ1	XinJ2	GuangD	GuangX	HaiN	Dianchi	GeJ	YiM	HeiJ	GuiZ	SiC	Tibet	
	0.031	0.391	0.003	0.217	0.002	0.092	0.175	0.061	0.077	0.023	0.001	0.001	0.013	0.001	0.001	0.001	0.001	0.002	HuB
0.155		0.381	0.105	0.346	0.008	0.289	0.417	0.478	0.392	0.056	0.021	0.136	0.199	0.016	0.015	0.001	0.001	0.013	HeN
0.000	0.008		0.031	0.379	0.009	0.349	0.301	0.168	0.378	0.367	0.050	0.035	0.198	0.025	0.032	0.046	0.001	0.023	ZheJ
0.670	0.186	0.477		0.062	0.148	0.117	0.101	0.019	0.492	0.006	0.058	0.120	0.084	0.030	0.033	0.014	0.009	0.106	NeiM
0.025	0.000	0.000	0.345		0.003	0.336	0.245	0.264	0.307	0.253	0.002	0.011	0.083	0.001	0.002	0.001	0.001	0.002	ShanX
0.408	0.238	0.366	0.175	0.275		0.015	0.047	0.005	0.093	0.001	0.001	0.001	0.003	0.001	0.001	0.001	0.001	0.007	JiL
0.107	0.000	0.000	0.145	0.000	0.208		0.301	0.382	0.374	0.089	0.017	0.180	0.261	0.036	0.016	0.017	0.001	0.009	QingH
0.115	0.000	0.000	0.994	0.000	0.293	0.000		0.361	0.429	0.099	0.145	0.431	0.474	0.106	0.161	0.117	0.005	0.111	XinJ1
0.168	0.000	0.086	0.714	0.040	0.356	0.000	0.000		0.504	0.017	0.054	0.038	0.097	0.007	0.030	0.002	0.003	0.005	XinJ2
0.182	0.000	0.000	0.000	0.020	0.165	0.000	0.000	0.000		0.096	0.061	0.277	0.225	0.044	0.117	0.061	0.003	0.017	GuangD
0.152	0.147	0.000	0.551	0.015	0.449	0.114	0.200	0.278	0.147		0.001	0.001	0.016	0.001	0.001	0.001	0.001	0.001	GuangX
0.331	0.204	0.166	0.294	0.251	0.371	0.183	0.075	0.152	0.141	0.350		0.012	0.005	0.005	0.231	0.038	0.001	0.030	HaiN
0.410	0.075	0.229	0.129	0.249	0.362	0.065	0.047	0.185	0.027	0.386	0.189		0.201	0.198	0.110	0.025	0.005	0.111	Dianchi_YunN
0.279	0.073	0.100	0.217	0.112	0.331	0.007	0.017	0.159	0.075	0.240	0.187	0.041		0.185	0.024	0.106	0.001	0.138	GeJ_YunN
0.461	0.233	0.307	0.332	0.329	0.464	0.191	0.189	0.301	0.212	0.448	0.194	0.035	0.038		0.033	0.049	0.001	0.259	YiM_YunN
0.371	0.183	0.206	0.230	0.273	0.379	0.175	0.088	0.172	0.105	0.364	0.019	0.070	0.143	0.105		0.063	0.013	0.195	HeiJ_YunN
0.449	0.224	0.153	0.385	0.268	0.524	0.188	0.175	0.293	0.149	0.330	0.120	0.164	0.088	0.138	0.090		0.001	0.013	GuiZ
0.869	0.745	0.775	0.779	0.779	0.836	0.706	0.769	0.795	0.671	0.840	0.445	0.534	0.592	0.524	0.244	0.591		0.020	SiC
0.845	0.617	0.655	0.674	0.690	0.806	0.531	0.620	0.731	0.504	0.801	0.310	0.224	0.258	0.058	0.088	0.339	0.433		Tibet
