# Supplementary material for: Historical Differentiation and Recent Hybridization in Natural Populations of the Nematode-Trapping Fungus Arthrobotrys oligospora in China
Source: Microorganisms. 2021 Sep 9;9(9):1919. doi: 10.3390/microorganisms9091919 (PMC8465350; doi:10.3390/microorganisms9091919)
Supplement: Supplementary file 1 [file microorganisms-09-01919-s001.zip › Table S4 Pairwise differentiations A. oligospora isolates from 19 geographic populations in China based on combined dataset..pdf]

Table S4 Pairwise differentiations *A. oligospora* isolates from 19 geographic populations in China based on combined dataset.

HuB	HeN	ZheJ	NeiM	ShanX	JiL	QingH	XinJ1	XinJ2	GuangD	GuangX	HaiN	Dianchi	GeJ	YiM	HeiJ	GuiZ	SiC	Tibet	
	0.080	0.270	0.010	0.030	0.010	0.170	0.090	0.040	0.040	0.030	0.010	0.010	0.010	0.010	0.010	0.010	0.010	0.010	HuB
0.047		0.400	0.010	0.090	0.010	0.320	0.310	0.040	0.520	0.010	0.010	0.090	0.050	0.010	0.010	0.010	0.010	0.030	HeN
0.030	0.000		0.020	0.120	0.010	0.450	0.090	0.030	0.430	0.010	0.010	0.120	0.060	0.020	0.020	0.010	0.010	0.050	ZheJ
0.339	0.295	0.232		0.010	0.010	0.020	0.040	0.010	0.030	0.010	0.010	0.020	0.030	0.010	0.010	0.010	0.010	0.040	NeiM
0.054	0.059	0.073	0.255		0.010	0.090	0.280	0.050	0.070	0.010	0.010	0.010	0.040	0.010	0.010	0.010	0.010	0.020	ShanX
0.277	0.269	0.191	0.146	0.266		0.010	0.010	0.010	0.010	0.010	0.010	0.010	0.010	0.010	0.010	0.010	0.010	0.010	JiL
0.032	0.000	0.000	0.198	0.041	0.172		0.110	0.060	0.330	0.010	0.010	0.270	0.180	0.010	0.020	0.010	0.010	0.040	QingH
0.102	0.046	0.160	0.566	0.024	0.483	0.072		0.350	0.060	0.010	0.010	0.200	0.030	0.010	0.020	0.010	0.010	0.120	XinJ1
0.104	0.074	0.138	0.415	0.090	0.397	0.087	0.000		0.040	0.010	0.010	0.080	0.050	0.020	0.010	0.010	0.010	0.040	XinJ2
0.087	0.000	0.000	0.236	0.092	0.190	0.010	0.151	0.103		0.020	0.020	0.130	0.030	0.020	0.020	0.010	0.020	0.020	GuangD
0.060	0.121	0.110	0.380	0.094	0.316	0.094	0.246	0.213	0.146		0.010	0.010	0.010	0.010	0.010	0.010	0.010	0.010	GuangX
0.384	0.344	0.236	0.357	0.377	0.386	0.274	0.383	0.363	0.282	0.383		0.010	0.010	0.010	0.020	0.010	0.010	0.010	HaiN
0.175	0.080	0.100	0.223	0.149	0.270	0.017	0.085	0.092	0.085	0.191	0.233		0.120	0.010	0.010	0.010	0.010	0.090	Dianchi
0.196	0.148	0.114	0.229	0.140	0.272	0.038	0.175	0.178	0.146	0.211	0.254	0.041		0.030	0.020	0.010	0.010	0.210	GeJ
0.348	0.286	0.259	0.358	0.329	0.388	0.203	0.348	0.330	0.273	0.349	0.276	0.140	0.082		0.010	0.010	0.010	0.360	YiM
0.274	0.204	0.129	0.264	0.268	0.308	0.136	0.253	0.259	0.167	0.269	0.119	0.092	0.133	0.165		0.020	0.010	0.110	HeiJ
0.495	0.437	0.317	0.511	0.494	0.538	0.361	0.534	0.497	0.388	0.495	0.162	0.295	0.316	0.280	0.146		0.010	0.030	GuiZ
0.569	0.539	0.507	0.630	0.554	0.651	0.442	0.609	0.554	0.512	0.566	0.357	0.309	0.373	0.398	0.217	0.512		0.030	SiC
0.533	0.467	0.409	0.586	0.497	0.633	0.316	0.563	0.510	0.431	0.530	0.302	0.180	0.114	0.000	0.128	0.353	0.396		Tibet
