# Supplementary figures and images for: Historical Differentiation and Recent Hybridization in Natural Populations of the Nematode-Trapping Fungus Arthrobotrys oligospora in China
Source: Microorganisms. 2021 Sep 9;9(9):1919. doi: 10.3390/microorganisms9091919 (PMC8465350; doi:10.3390/microorganisms9091919)

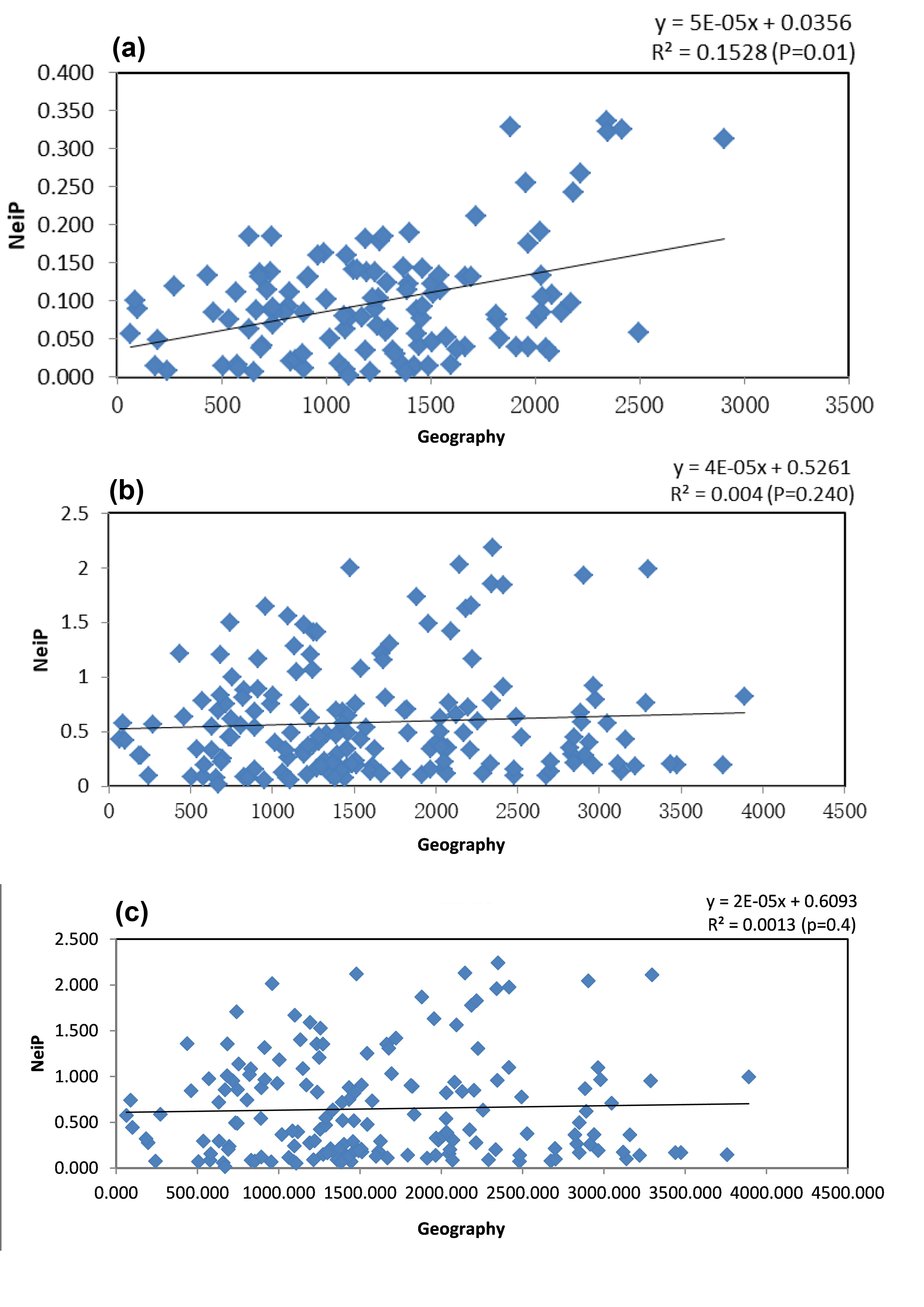

Supplement: Supplementary file 1 [file microorganisms-09-01919-s001.zip › Figure S2 Mantel tests.tif]

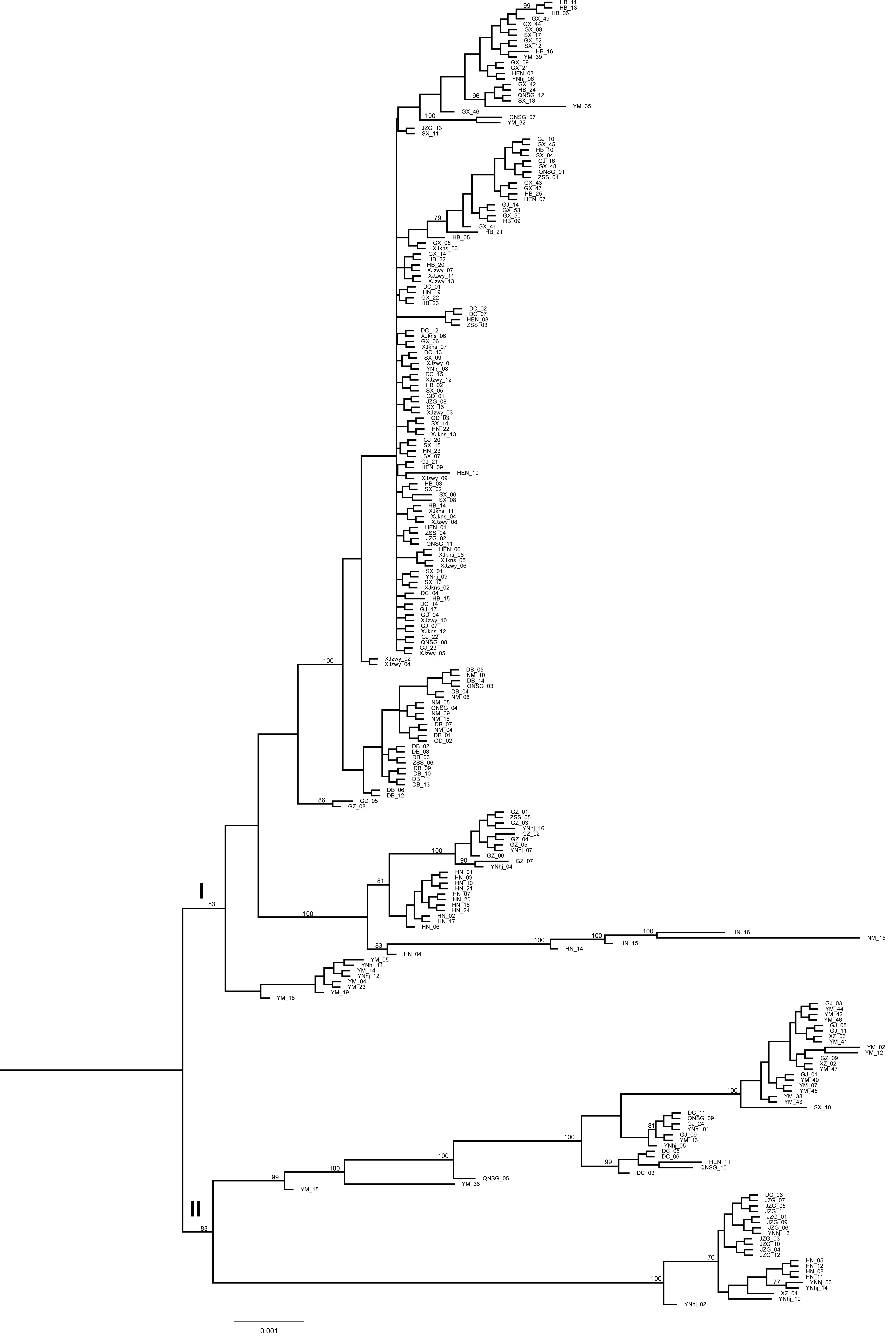

Supplement: Supplementary file 1 [file microorganisms-09-01919-s001.zip › Figure S4 Bayesian phylogeny for the MLST SNPs.jpg]
